# Single-nucleotide polymorphism rs2910829 in *PDE4D* is related to stroke susceptibility in Chinese populations: The results of a meta-analysis

**DOI:** 10.1515/biol-2022-0818

**Published:** 2024-02-23

**Authors:** Guiying Zhang, Xuelei Tang, Qifu Li, Rong Lin

**Affiliations:** Department of Biology, Hainan Medical University, Haikou, China; Department of Neurology, The First Affiliated Hospital of Hainan Medical University, Haikou, China; Center of Forensic Medicine of Hainan Medical University, Hainan Provincial Academician Workstation (Tropical Forensic Medicine), Hainan Provincial Tropical Forensic Engineering Research Center, Haikou, China

**Keywords:** PDE4D, rs2910829, polymorphism, ischaemic stroke, large artery atherosclerosis stroke, meta-analysis

## Abstract

Stroke is a debilitating condition that often leads to disability and death. The increasing prevalence of stroke has drawn worldwide attention. Extensive evidence indicates a crucial role of genetic determinants in the occurrence and perpetuation of stroke. An Icelandic study identified a significant correlation of the phosphodiesterase 4D (*PDE4D*) single-nucleotide polymorphism (SNP) rs2910829 with stroke susceptibility. However, subsequent studies reported in Chinese populations were contradictory. We implemented a meta-analysis to inspect whether SNP rs2910829 is related to stroke susceptibility in Chinese populations and subsequently performed an *in silico* analysis to predict its potential functions. Finally, we analysed data from 24 studies comprising 7,484 Chinese stroke patients and 7,962 control individuals. Compared with the CC genotype, the TT genotype was associated with increased susceptibility to stroke (pooled odds ratio [OR] 1.28, 95% confidence interval [CI] 1.13–1.46, *P* < 0.001), whereas the CT genotype was not. Correspondingly, a significant association was detected under the recessive model (TT vs CT + CC: OR 1.30, 95% CI 1.15–1.47, *P* < 0.001). Similar results were obtained in large artery atherosclerosis (LAA) stroke but not in small vessel stroke. Bioinformatics analysis also revealed that SNP rs2910829 and its linked SNPs might be implicated in transcriptional regulation. This meta-analysis reveals significant relationships between the *PDE4D* SNP rs2910829 and susceptibility to stroke and subtype-LAA stroke in Chinese individuals, and further investigations are warranted to evaluate this effect.

## 1 Introduction

Stroke is a leading cause of disability and death in adults [1]. It poses a significant health burden worldwide, including in China. In China, the prevalence of stroke is on the rise, and it is estimated that approximately 13 million patients suffer from stroke. The incidence of first stroke among adults aged 40–74 years increased by 8.3% per year from 2002 to 2013 [2].

Stroke caused by monogenic genetic diseases accounts for a very small proportion, perhaps less than 5%, and this proportion is larger in young stroke patients. The vast majority of strokes are complex diseases caused by the combined effects of multiple genetic and environmental factors. In 2002, using a genome-wide search approach, the deCODE group successfully mapped a candidate region on chromosome 5q12 for stroke in Icelandic families [3]. Fine mapping of the locus revealed that phosphodiesterase 4D (*PDE4D*, OMIM: 600129) may be a susceptibility gene, and subsequent association analysis in an Icelandic stroke cohort identified several significant single-nucleotide polymorphisms (SNPs) and haplotypes in *PDE4D* [4].

PDE4D is a phosphodiesterase that can specifically hydrolyze cyclic adenosine monophosphate (cAMP) [5]. Endothelial cell integrity, smooth muscle cell function, and inflammation are important in the progression of atherosclerosis [6], large artery atherosclerosis (LAA), and cardioembolic (CE) stroke [7,8]. The long-term elevation of cAMP levels increases the expression of specific PDE4D isoforms through cAMP-dependent transcription factors in human endothelial cells [9], and raises histone levels of *PDE4D* promoter sequences in activated smooth muscle cells, which may cause atherosclerosis [10,11]. The activation and proliferation of synthetic smooth muscle cells in the intimal layer of the vessel wall promotes the formation of atherosclerosis. PDE4D is involved in the regulation of cAMP signalling in inflammation. PDE4 inhibitors lower the expression of inflammatory cytokines and the migration of inflammatory cells [12,13]. Inflammatory processes increase the vulnerability of cardiac tissue and play a large part in the development and persistence of atrial fibrillation [14,15]. Atrial fibrillation is one of the major risk factors for ischaemic stroke (IS) [16], and concurrent inflammation may further increase the risk [17]. Therefore, PDE4D may be involved in the pathogenesis of stroke, particularly the pathogenesis of LAA and CE stroke.

In the past decade, many studies have attempted to replicate the findings of the deCODE group. In Chinese populations, SNP rs2910829 (SNP87) in *PDE4D* is a popular research topic. However, the studies have shown conflicting results. For instance, in Chinese Han populations, Lin et al. [18] and Xu [19] took the lead in detecting SNP rs2910829, but they did not find a significant association of SNP rs2910829 with susceptibility to early onset IS or IS, but Wang and Zhang [20] observed a significant association of SNP rs2910829 with stroke susceptibility.

Therefore, to clarify the relationship between SNP rs2910829 and susceptibility to stroke in Chinese populations, we conducted the current meta-analysis. The functional outcomes of SNP rs2910829 are not yet clear, so preliminary predictions were next made using bioinformatics analysis in the present work. In summary, stroke is prevalent around the world, including in China and its genetic determinants are poorly understood. We addressed this issue and hope that our results will be helpful in the prevention, diagnosis, and treatment of stroke.

## 2 Methods

This study followed the Preferred Reporting Items for Systematic Reviews and Meta-Analyses (PRISMA) 2020 statement [21]. The PRISMA checklist is available in Supplementary Materials.

### 2.1 Literature search

A comprehensive literature search without any language restriction was performed on several databases including PubMed, Embase, ISI Web of Science, Weipu, China National Knowledge Infrastructure (CNKI), Chinese Biomedical (CBM), and Wanfang from inception through August 18, 2022. The search terms used were “phosphodiesterase 4D,” “PDE4D,” “SNP87,” “SNP 87,” “rs2910829,” “stroke,” “cerebral infarction,” “ischaemic stroke,” “ischemic stroke,” “cerebrovascular disease,” and their synonyms. The references of the included literature, as well as relevant meta-analyses and reviews, were also screened to determine whether there were potential studies for inclusion.

### 2.2 Study selection criteria

Studies that met the following criteria were included in the analysis: (1) case‒control, nested case‒control, or cohort studies; (2) evaluating the association of *PDE4D* (NG_027957.2) SNP rs2910829 with stroke susceptibility in Chinese populations; and (3) using verified genotyping techniques. Reviews, editorials, case reports, case-only studies, family-based studies, and other articles without primary research findings were excluded.

### 2.3 Data extraction and quality assessment

The data extracted from each qualified study included the first author’s name, year of publication, ethnicity of the subjects studied, sample size of patients and control individuals, average age of participants, genotyping methods, matching criteria for control individuals, stroke subtypes (if reported), and distribution of genotypes and alleles. Additionally, if the full text or information needed was unavailable, it would be requested from the authors by email and/or phone.

The methodological quality of each individual study was assessed using the Newcastle‒Ottawa Scale (NOS) [22]. A study can be awarded a maximum of nine scores. Higher scores indicate better methodological quality of the included studies. Studies scoring less than 4 points were excluded from the analysis due to poor methodological quality.

Two reviewers (G.Z. and X.T.) independently screened the literature, collected the data, and evaluated the study qualities. The discrepancies were settled by checking and discussing with the senior author (R.L.).

### 2.4 Statistical analyses

Hardy–Weinberg equilibrium (HWE) among the control individuals was assessed by a *χ* ^2^ test. STATA 11.0 software (Stata Corporation, College Station, TX) was used for the statistical analysis. Odds ratios (ORs) and corresponding 95% confidence intervals (CIs) were calculated to assess the strength of the association between SNP rs2910829 and stroke susceptibility for comparisons of two different genotypes (CT vs CC and TT vs CC), as well as under dominant (CT + TT vs CC), additive (T vs C), and recessive (TT vs CT + CC) genetic models.

The heterogeneity between studies was checked by the *χ* ^2^-based *Q*-test and *I* ^2^ test. *P* < 0.10 indicated significant heterogeneity across studies, and a random-effects model using the DerSimonian‒Laird method was chosen for the data analysis; otherwise, a fixed-effects model using the Mantel‒Haenszel method was selected. The *I* ^2^ values of <25, 25–50, 50–75, and 75–100% were regarded as no, moderate, large, and extreme heterogeneity, respectively. Meta-regression analysis was conducted to investigate the potential sources of heterogeneity. The publication bias was assessed with Begg’s test and Egger’s linear regression test and visualized with Begg’s funnel and Egger’s publication bias plots.

Sensitivity analyses were carried out to assess the stability of the results. Each study was evaluated using the leave-one-out method, and pooled estimates for the remaining studies were calculated. Sensitivity analyses were also conducted by omitting studies if the genotype frequency of the control individuals deviated from HWE.

### 2.5 Functional annotation

The HaploReg (http://pubs.broadinstitute.org/mammals/haploreg/haploreg.php) and RegulomeDB (http://regulomedb.org/) databases were utilized to investigate the potential biological functions of SNP rs2910829. Both databases can supply functional annotations for regulatory characteristics of genetic variants situated in noncoding regions.

## 3 Results

### 3.1 Eligible studies

**Figure 1:**
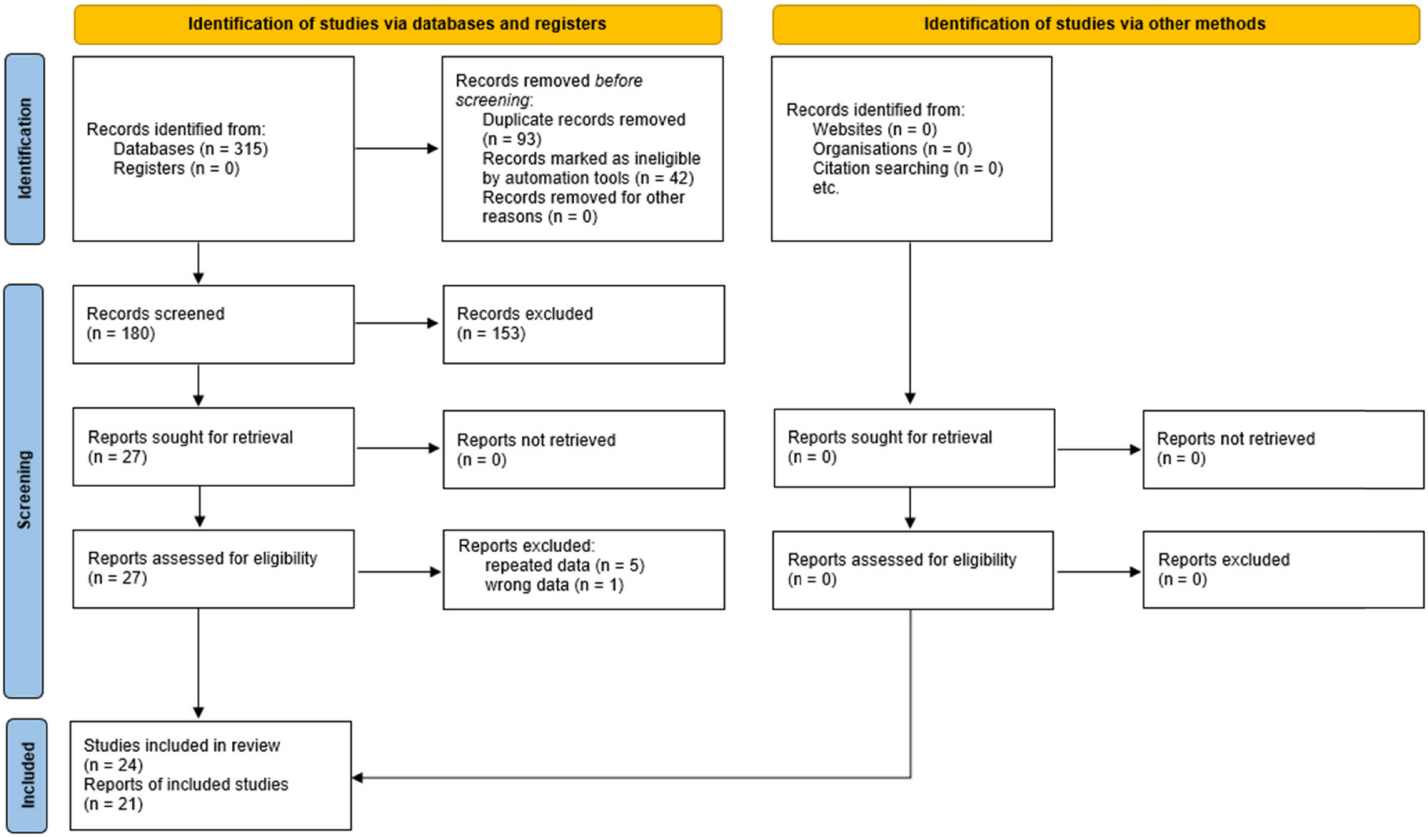
PRISMA 2020 flow diagram of the literature screening process.

As illustrated in Figure 1, a total of 27 articles met the inclusion criteria. After reading the full texts carefully, six were excluded: five that reported on overlapping populations [23–27], as well as one whose data were obviously wrong [28]. Three articles assessed correlations in independent populations, so each article was considered as two separate studies [29–31]. Finally, 24 studies (in 21 articles involving 7,484 stroke patients and 7,962 control individuals) were enrolled in the meta-analysis on SNP rs2910829 and stroke susceptibility (Table 1 and Table S1) [18–20,29–46]. Each study design was case‒control. The genotype frequencies of four studies [36–38,46] were not consistent with HWE expectations in control individuals (Table S1). The NOS results showed that each included study received scores of no less than 4 for methodological quality assessment, with an average score of 6.5 (Table 1).

**Table 1:** Main characteristics of selected studies in the meta-analysis of the association between SNP rs2910829 and stroke susceptibility

First author	Year	Ethnicity	Sample size		Mean age ± SD (year)		Genotyping method	Matching criteria for controls	Phenotype	NOS
			Cases	Controls	Cases	Controls				
Lin HF	2007	Chinese Han	180	210			TaqMan	Age and gender	Early onset IS	6
Xu SL	2008	Chinese Han	116	110	65.9 ± 12.4	65.1 ± 12.7	PCR-RFLP	Age and gender	LAA and SVD	6
Hsieh MS	2009	Chinese Han	108	280	70 ± 11	63 ± 23	TaqMan	Age and gender	IS	5
Xue H	2009	Chinese Han	639	887	60.8 ± 9.2	60.7 ± 8.2	PCR-RFLP	Age, gender, and geographical region	LAA, SVD, and HS	7
Sun Y	2009	Chinese Han	646	761	73.20 ± 9.41	73.2 ± 7.30	Sanger sequencing	Age, gender, and BMI	IS	8
Wang SR	2009	Chinese Han	122	44	62.57 ± 13.49	58.61 ± 17.55	PCR-LDR	Age, gender, and BMI	LAA, SVD, and HS	7
He Y	2012	Chinese Han	400	400	61 ± 10	58 ± 10	PCR-RFLP	Age and gender	IS	7
Zhang XN	2012	Chinese Han	116	118	61.6 ± 10.6	61.6 ± 10.1	PCR-RFLP	Age and gender	IS	6
Li C	2012	Chinese Han	440	486	66.58 ± 8.40	66.10 ± 5.18	PCR-RFLP	Age and gender	IS	6
Li N	2012	Chinese Han	371	371	63.88 ± 7.36	62.87 ± 7.57	PCR-RFLP	Age, gender, and hypercholesterolemia	LAA	7
Zhao J	2012	Chinese Han	682	598	62.09 ± 9.43	61.84 ± 10.12	PCR-RFLP	Age, gender, and BMI	LAA	8
He Y	2013	Chinese Han	186	232	36.5 ± 6.4	36.8 ± 6.8	PCR-RFLP	Age and gender	Early onset IS	6
Wang RX	2014	Chinese Han	245	209	62.88 ± 8.75	61.21 ± 7.56	PCR-RFLP	Age and gender	LAA	6
Ma J	2014	Chinese Han	189	194			PCR-RFLP	Age and gender	IS	6
Shao M	2015	Chinese Han	459	462	68.56 ± 10.97	63.82 ± 9.22	MALDI-TOF	Gender, smoking, and drinking	LAA and SVD	7
Shi JP	2015	Chinese Han	126	128	60.9 ± 9.7	62.1 ± 9.4	PCR-RFLP	Age and gender	IS	6
Feng XW	2015	Chinese Han	168	172	65.5 ± 4.7	66.1 ± 5.3	PCR-RFLP	Age, gender, and drinking history	IS	7
Yuan JG	2016	Chinese Han	183	183	60.2 ± 10.6	60.1 ± 11.3	PCR-RFLP	Age and gender	IS	5
Wang X	2017	Chinese Han	610	618	65.8 ± 15.2	66.5 ± 16.1	PCR-RFLP	Age and gender	IS	7
Zhang L	2019	Chinese Han	881	892	64.5 ± 14.7	65.6 ± 15.3	PCR-RFLP	Age, gender, and BMI	IS	8
Yue X	2019	Chinese Han	193	200	33.2 ± 12.8	31.1 ± 17.9	Semiconductor sequencing	Age, gender, drinking history, and diabetes history	Early onset IS	6
Zhang XN	2012	Chinese Uyghur	110	102	61.5 ± 9.9	58.2 ± 9.4	PCR-RFLP	Age and gender	IS	6
Ma J	2014	Chinese Uyghur	184	183			PCR-RFLP	Age and gender	IS	6
Shi JP	2015	Chinese Mongolian	130	122	60.87 ± 8.1	59.13 ± 8.9	PCR-RFLP	Age and gender	IS	6

PCR-RFLP: polymerase chain reaction-restriction fragment length polymorphism; PCR-LDR: polymerase chain reaction ligase detection reaction; MALDI-TOF: matrix-assisted laser desorption/ionization time-of-flight; BMI: body mass index; IS: ischaemic stroke; LAA: large artery atherosclerosis; SVD: cerebral small vessel disease; HS: haemorrhagic stroke; NOS, Newcastle–Ottawa Scale.

### 3.2 SNP rs2910829 and stroke susceptibility

**Figure 2:**
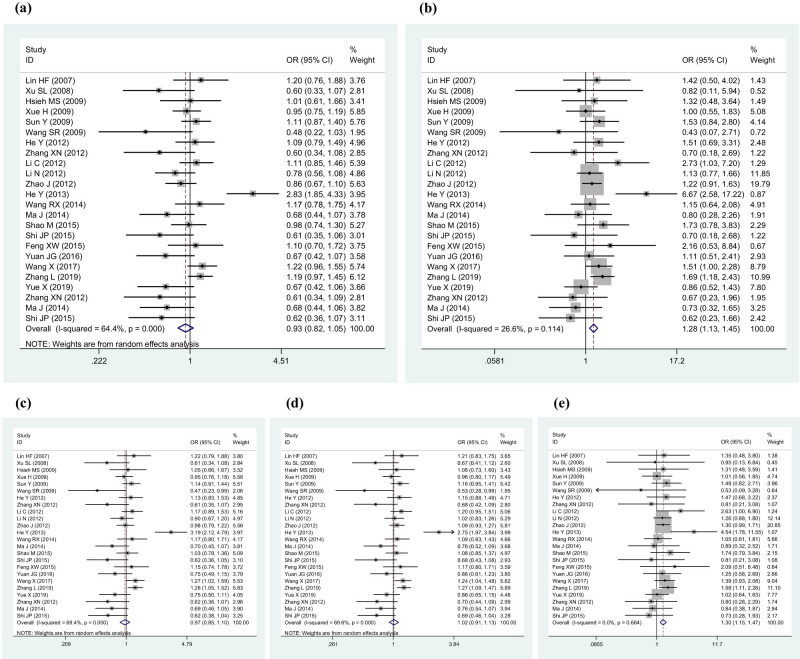
Forest plot for the association between stroke susceptibility and SNP rs2910829: (a) (CT vs CC) (random effects), (b) (TT vs CC) (fixed effects), (c) under the dominant model (CT + TT vs CC) (random effects), (d) under the additive model (T vs C) (random effects), and (e) under the recessive model (TT vs CT + CC) (fixed effects).

The results of the meta-analysis of the association between SNP rs2910829 and stroke susceptibility are summarized in Table 2 and Figure 2. As shown, compared with the CC genotype, the TT genotype was correlated with a greater susceptibility to stroke (OR 1.28, 95% CI 1.13–1.46, *P* < 0.001) with moderate heterogeneity (*I* ^2^ = 26.6%, *P* = 0.114), whereas the CT genotype was not. Correspondingly, a significant correlation was observed under the recessive model (TT vs CT + CC: OR 1.30, 95% CI 1.15–1.47, *P* < 0.001). No heterogeneity was found across all studies under the recessive model (*I* ^2^ = 0.0%, *P* = 0.664).

**Table 2:** Meta-analysis of the association between SNP rs2910829 and stroke susceptibility

	Pooled OR (95% CI)	*P*_OR_	*I* ^2^	*P*_H_	Statistical model
CT vs CC	0.93 (0.82–1.05)	0.247	64.4%	**<0.001**	Random
TT vs CC	1.28 (1.13–1.46)	**<0.001**	26.6%	0.114	Fixed
Dominant	0.97 (0.85–1.10)	0.620	69.4%	**<0.001**	Random
Additive	1.02 (0.92–1.13)	0.770	69.6%	**<0.001**	Random
Recessive	1.30 (1.15–1.47)	**<0.001**	0.0%	0.664	Fixed

*P*_OR_ and *P*_H_ are *P* values for odds ratio and heterogeneity, respectively. *P*_OR_ values significant at *P* < 0.05 and *P*_H_ values significant at *P* < 0.10 are shown in bold.

Three of those 24 studies focused on early-onset IS [18,39,46], and when we excluded them, the findings were basically unchanged. The phenotype of the other two studies was stroke [20,33]. The results remained similar after we excluded those three studies on early-onset IS and haemorrhagic stroke (HS) patients from the two studies on stroke (Tables S2–S4 and Figures S1–S5).

Next, we performed an analysis of other stroke subtypes. Eight studies were finally selected for the meta-analysis of LAA stroke, and six studies were selected for small vessel stroke (Tables S5 and S6). There was only one study for CE stroke, two studies for combined CE and LAA stroke, and two studies for HS. Therefore, there were not enough data for meta-analyses of these stroke subtypes. The results showed that SNP rs2910829 was related to susceptibility to LAA stroke (Table S7 and Figures S6–S10) but not to susceptibility to small vessel stroke (Table S8 and Figures S11–S15). The results of LAA stroke were similar to those of stroke: (1) compared with the CC genotype, the TT genotype was correlated with a greater susceptibility to LAA stroke (OR 1.22, 95% CI 1.00–1.49, *P* = 0.045) without heterogeneity (*I* ^2^ = 0.0%, *P* = 0.810), whereas the CT genotype was not and (2) a significant correlation was observed under the recessive model (TT vs CT + CC: OR 1.29, 95% CI 1.07–1.55, *P* = 0.008) without heterogeneity (*I* ^2^ = 0.0%, *P* = 0.847).

### 3.3 Sensitivity analyses

All the OR values were not substantially altered after conducting sensitivity analyses via the leave-one-out method (Figures S16–S20) and by excluding the four HWE-violating studies (Table S9).

### 3.4 Publication bias

**Figure 3:**
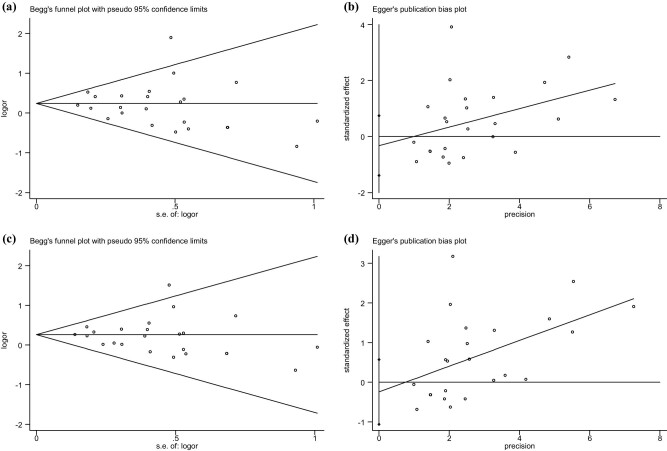
Begg’s funnel and Egger’s publication bias plots for the association between stroke susceptibility and SNP rs2910829: (a) (TT vs CC) (Begg’s *P* = 0.286), (b) (TT vs CC) (Egger’s *P* = 0.536), (c) under the recessive model (TT vs CT + CC) (Begg’s *P* = 0.472), and (d) under the recessive model (TT vs CT + CC) (Egger’s *P* = 0.541).

Begg’s funnel and Egger’s publication bias plots for positive results are shown in Figure 3. There was no significant publication bias.

### 3.5 Functional annotation

According to HaploReg v4.1, two SNPs (rs10939837 and rs6449458) were in strong linkage disequilibrium (LD) with SNP rs2910829 (*r* ^2^ ≥ 0.8) in the East Asian (CHB, JPT, and CHS) population (Table S10). All three SNPs are located in the intron regions of *PDE4D* and might alter transcriptional regulatory element activity. Specifically, SNPs rs10939837, rs6449458, and rs2910829 altered 10, 1, and 3 regulatory motifs, respectively.

Based on RegulomeDB v2.1, all three SNPs were linked to the expression of the protocadherin beta 19 pseudogene (*PCDHB19P*), which is located approximately 657.8 kb upstream of *PDE4D*, in frontal cortex tissue (Table S11). For SNP rs2910829, the TT genotype was associated with the highest expression level of *PCDHB19P*, followed by the CT genotype and then the CC genotype (*P* = 3.53 × 10^−5^). SNPs rs10939837 and rs2910829 might affect the expression of prostate androgen-regulated transcript 1 *(PART1)*, having a 5ʹ end that overlaps with the 5ʹ end of *PDE4D*, in esophageal muscularis mucosa tissue. The genomic region containing SNP rs10939837 was a DNase hypersensitive region detected in brain tissue. SNPs rs10939837 and rs2910829 changed the regulatory motifs to eight proteins and one protein, respectively. Among the three SNPs, SNP rs10939837 had the lowest RegulomeDB rank (Rank = 1f), which might indicate the strongest functional significance. The RegulomeDB rank ranges from 1a to 7, with a lower rank indicating an increased probability of having a regulatory function. Both databases showed that these three SNPs might be involved in transcriptional regulation. However, further investigation is needed to determine which of them is the real causative variant.

## 4 Discussion

The present meta-analysis revealed that SNP rs2910829 was associated with susceptibility to stroke, especially LAA stroke, in Chinese populations. To our knowledge, thus far, this study is the largest meta-analysis of the relationship between SNP rs2910829 and stroke susceptibility in Chinese populations, and few subtype analyses of stroke have been performed in meta-analyses of the relationship between SNP rs2910829 and stroke susceptibility. Stroke subtypes were analysed in the present meta-analysis.

A total of nine meta-analysis reports on SNP rs2910829 and stroke risk have been documented up to 2022 (Table S12) [47–55]. In 2006, a meta-analysis of nine studies on 3,808 stroke patients and 4,377 control individuals indicated a significant association between stroke risk and SNP rs2910829 (pooled *P* = 0.002) [47]. However, the subsequent seven meta-analyses all showed that SNP rs2910829 was not associated with IS [48–54]. Among the eight meta-analyses mentioned above, one [49] was conducted only among Asians, one [52] was conducted only among South Asians, and four [50,51,53,54] were also subgrouped for Asians. However, consistent nonsignificant associations in Asians were shown in the six meta-analyses. In 2017, a meta-analysis [55] of 26 studies on 10,529 IS patients and 12,223 control individuals revealed no statistically significant associations with IS for SNP rs2910829 in the overall population [55]. Nevertheless, in the subgroup analysis, a significant correlation was detected under the recessive model (OR 1.15, 95% CI 1.01–1.30, *P* = 0.030; *I* ^2^ = 0.0%, *P* = 0.900) among Asians, which was very similar to our results in Chinese individuals but not among Caucasians. Among the meta-analyses in Asians, the meta-analysis conducted by Wei et al. [55] included the largest number of studies and combined sample size, followed by the meta-analysis conducted by Liang et al. [53]. Unfortunately, Wei et al. [55] did not conduct further analysis of stroke subtypes. Among the nine meta-analyses, only one [48] included further analysis of stroke subtypes. The above meta-analysis showed that SNP rs2910829 was not associated with either IS or its subtypes [48]. Of note, the majority of subjects in that meta-analysis were Caucasian.

Our current meta-analysis was conducted only among Chinese people and was not extended to other Asian populations or Caucasians, which is a limitation of this study. However, several previous studies have conducted meta-analyses with Asians and Caucasians, while no other detailed meta-analysis has been conducted with Chinese individuals. The effect of SNP rs2910829 on stroke is different between Asians and Caucasians, and its effect in Chinese individuals also needs to be addressed. Here, we conducted a specialized meta-analysis of Chinese individuals to clarify the role of SNP rs2910829 in stroke in Chinese individuals.

As stated in the introduction, PDE4D may be implicated in the pathogenesis of stroke, particularly the pathogenesis of LAA and CE stroke. The deCODE group observed the association of *PDE4D* variants with stroke, especially with CE and LAA stroke [4]. SNP rs2910829 was reported to be associated with IS susceptibility, particularly susceptibility to combined CE and LAA stroke. The results of our current meta-analysis were basically consistent with those of previous studies. Unfortunately, no additional studies have explored the association of SNP rs2910829 with susceptibility to CE stroke or combined CE and LAA stroke.

*In silico* analysis showed that the TT genotype of SNP rs2910829 was associated with the highest expression level of *PCDHB19P* in frontal cortex tissue. The present meta-analysis also showed that the TT genotype was associated with an increased susceptibility to stroke, especially LAA stroke. To date, no studies have predicted the functions of SNP rs2910829 and other variants in strong LD with it, and no corresponding functional studies have been conducted on them. The present study preliminarily predicted their functions. Therefore, further functional studies are needed to determine whether they are true pathogenic variants.

## 5 Conclusions

This meta-analysis suggests that SNP rs2910829 in *PDE4D* may contribute to stroke susceptibility, especially LAA stroke susceptibility, in Chinese individuals. This study provides a better understanding of the association of *PDE4D* SNP rs2910829 with stroke susceptibility in Chinese individuals. Preliminary bioinformatics analysis also indicates that SNP rs2910829 and its linked SNPs may take part in transcriptional regulation. In the future, well-designed epidemiologic studies will help illuminate this impact on stroke, especially on CE and LAA stroke. The potential mechanisms linking the variants to the disease also require further functional studies.

## Supplementary Material

Supplementary material
