# Supplementary material for: Single-nucleotide polymorphism rs2910829 in PDE4D is related to stroke susceptibility in Chinese populations: The results of a meta-analysis
Source: Open Life Sci. 2024 Feb 23;19(1):20220818. doi: 10.1515/biol-2022-0818 (PMC10921477; doi:10.1515/biol-2022-0818)
Supplement: Supplementary material [file biol-2022-0818-sm.pdf]

Supplementary material

Prisma 2020 checklist

Section and Topic	Item #	Checklist item	Location where item is reported
**TITLE**			
Title	1	Identify the report as a systematic review.	Page 1
**ABSTRACT**			
Abstract	2	See the PRISMA 2020 for Abstracts checklist.	Page 2
**INTRODUCTION**			
Rationale	3	Describe the rationale for the review in the context of existing knowledge.	Pages 4–5
Objectives	4	Provide an explicit statement of the objective(s) or question(s) the review addresses.	Pages 5–6
**METHODS**			
Eligibility criteria	5	Specify the inclusion and exclusion criteria for the review and how studies were grouped for the syntheses.	Pages 6–7
Information sources	6	Specify all databases, registers, websites, organisations, reference lists and other sources searched or consulted to identify studies. Specify the date when each source was last searched or consulted.	Page 6
Search strategy	7	Present the full search strategies for all databases, registers and websites, including any filters and limits used.	Page 6
Selection process	8	Specify the methods used to decide whether a study met the inclusion criteria of the review, including how many reviewers screened each record and each report retrieved, whether they worked independently, and if applicable, details of automation tools used in the process.	Pages 6–7
Data collection process	9	Specify the methods used to collect data from reports, including how many reviewers collected data from each report, whether they worked independently, any processes for obtaining or confirming data from study investigators, and if applicable, details of automation tools used in the process.	Page 7
Data items	10a	List and define all outcomes for which data were sought. Specify whether all results that were compatible with each outcome domain in each study were sought (e.g. for all measures, time points, analyses), and if not, the methods used to decide which results to collect.	Page 7
	10b	List and define all other variables for which data were sought (e.g. participant and intervention characteristics, funding sources). Describe any assumptions made about any missing or unclear information.	Page 7

(*Continued*)

(*Continued*)

Study risk of bias assessment	11	Specify the methods used to assess risk of bias in the included studies, including details of the tool(s) used, how many reviewers assessed each study and whether they worked independently, and if applicable, details of automation tools used in the process.	Page 7
Effect measures	12	Specify for each outcome the effect measure(s) (e.g. risk ratio, mean difference) used in the synthesis or presentation of results.	Pages 7–8
Synthesis methods	13a	Describe the processes used to decide which studies were eligible for each synthesis (e.g. tabulating the study intervention characteristics and comparing against the planned groups for each synthesis (item #5)).	Pages 6–7
	13b	Describe any methods required to prepare the data for presentation or synthesis, such as handling of missing summary statistics, or data conversions.	Pages 7–8
	13c	Describe any methods used to tabulate or visually display results of individual studies and syntheses.	Pages 7–8
	13d	Describe any methods used to synthesize results and provide a rationale for the choice(s). If meta-analysis was performed, describe the model(s), method(s) to identify the presence and extent of statistical heterogeneity, and software package(s) used.	Pages 7–8
	13e	Describe any methods used to explore possible causes of heterogeneity among study results (e.g. subgroup analysis, meta-regression).	Page 8
	13f	Describe any sensitivity analyses conducted to assess robustness of the synthesized results.	Page 8
Reporting bias assessment	14	Describe any methods used to assess risk of bias due to missing results in a synthesis (arising from reporting biases).	Page 8
Certainty assessment	15	Describe any methods used to assess certainty (or confidence) in the body of evidence for an outcome.	Page 8
**RESULTS**			
Study selection	16a	Describe the results of the search and selection process, from the number of records identified in the search to the number of studies included in the review, ideally using a flow diagram.	Page 9; Figure 1
	16b	Cite studies that might appear to meet the inclusion criteria, but which were excluded, and explain why they were excluded.	Page 9
Study characteristics	17	Cite each included study and present its characteristics.	Page 9; Table 1
Risk of bias in studies	18	Present assessments of risk of bias for each included study.	Page 9; Table 1
Results of individual studies	19	For all outcomes, present, for each study: (a) summary statistics for each group (where appropriate) and (b) an effect estimate and its precision (e.g. confidence/credible interval), ideally using structured tables or plots.	Figure 2; Figures S1–S15
Results of syntheses	20a	For each synthesis, briefly summarise the characteristics and risk of bias among contributing studies.	Pages 9–11; Table 2; Figure 2; Tables S4, S7–S9; Figures S1–S15

(*Continued*)

(*Continued*)

	20b	Present results of all statistical syntheses conducted. If meta-analysis was done, present for each the summary estimate and its precision (e.g. confidence/credible interval) and measures of statistical heterogeneity. If comparing groups, describe the direction of the effect.	Pages 9–11; Table 2; Figure 2; Tables S4, S7–S9; Figures S1–S15
	20c	Present results of all investigations of possible causes of heterogeneity among study results.	Pages 10–11
	20d	Present results of all sensitivity analyses conducted to assess the robustness of the synthesized results.	Page 11; Figures S16–S20; Table S9
Reporting biases	21	Present assessments of risk of bias due to missing results (arising from reporting biases) for each synthesis assessed.	Page 11; Figure 3
Certainty of evidence	22	Present assessments of certainty (or confidence) in the body of evidence for each outcome assessed.	Page 11; Figure 3; Figures S16–S20; Table S9
**DISCUSSION**			
Discussion	23a	Provide a general interpretation of the results in the context of other evidence.	Pages 12–14
	23b	Discuss any limitations of the evidence included in the review.	Pages 14–15
	23c	Discuss any limitations of the review processes used.	Pages 14–15
	23d	Discuss implications of the results for practice, policy, and future research.	Pages 14–15
**OTHER INFORMATION**			
Registration and protocol	24a	Provide registration information for the review, including register name and registration number, or state that the review was not registered.	Page 16
	24b	Indicate where the review protocol can be accessed, or state that a protocol was not prepared.	Page 16
	24c	Describe and explain any amendments to information provided at registration or in the protocol.	N/A
Support	25	Describe sources of financial or non-financial support for the review, and the role of the funders or sponsors in the review.	Page 16
Competing interests	26	Declare any competing interests of review authors.	Page 16
Availability of data, code and other materials	27	Report which of the following are publicly available and where they can be found: template data collection forms; data extracted from included studies; data used for all analyses; analytic code; any other materials used in the review.	Page 16

From: Page MJ, McKenzie JE, Bossuyt PM, Boutron I, Hoffmann TC, Mulrow CD, et al. The PRISMA 2020 statement: an updated guideline for reporting systematic reviews. BMJ 2021;372:n71. doi: 10.1136/bmj.n71. For more information, visit: http://www.prisma-statement.org/.

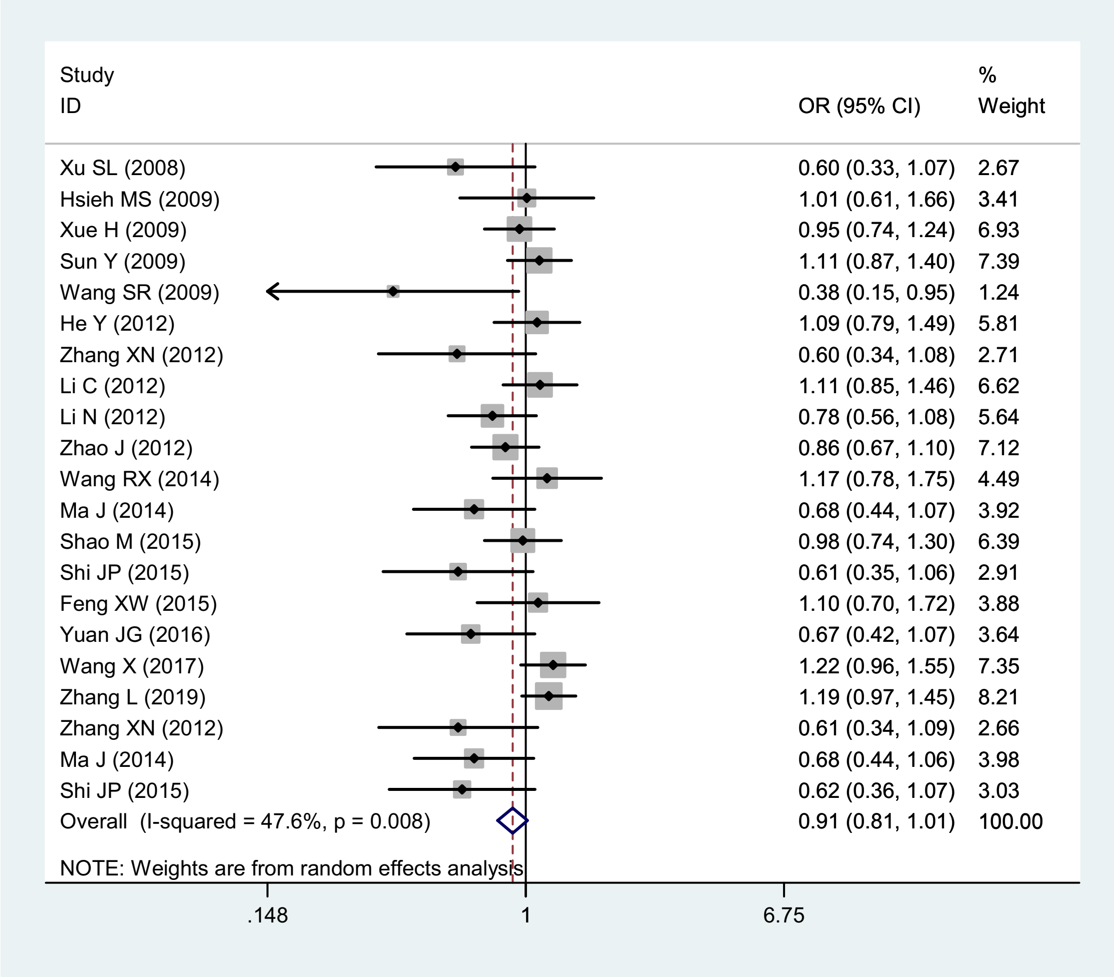

Figure S1: Forest plot for the association between stroke susceptibility and SNP rs2910829 (CT vs CC) (random effects) after excluding those 3 studies on early-onset ischemic stroke (Lin HF(2007), He Y(2013), and Yue X(2019)) and hemorrhagic stroke cases from two studies (Xue H(2009) and Wang SR(2009)).

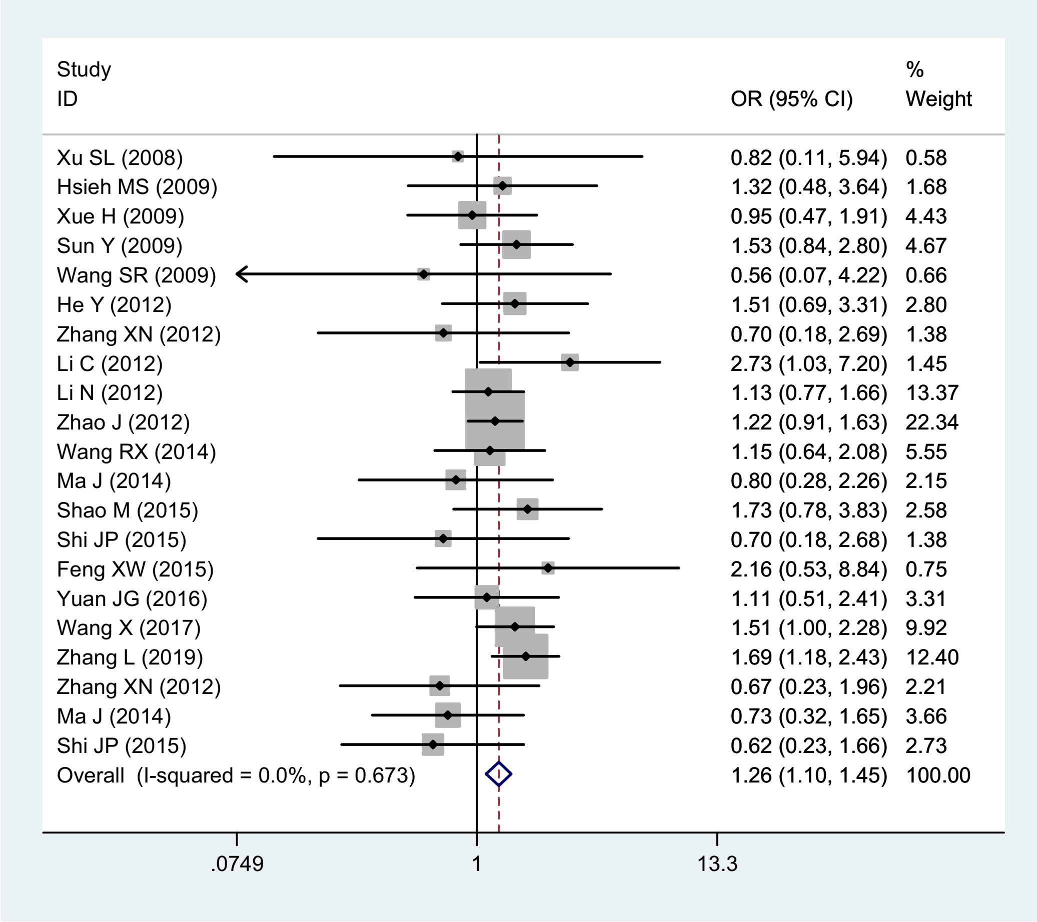

Figure S2: Forest plot for the association between stroke susceptibility and SNP rs2910829 (TT vs CC) (fixed effects) after excluding after excluding those 3 studies on early-onset ischemic stroke (Lin HF(2007), He Y(2013), and Yue X(2019)) and hemorrhagic stroke cases from 2 studies (Xue H(2009) and Wang SR(2009)).

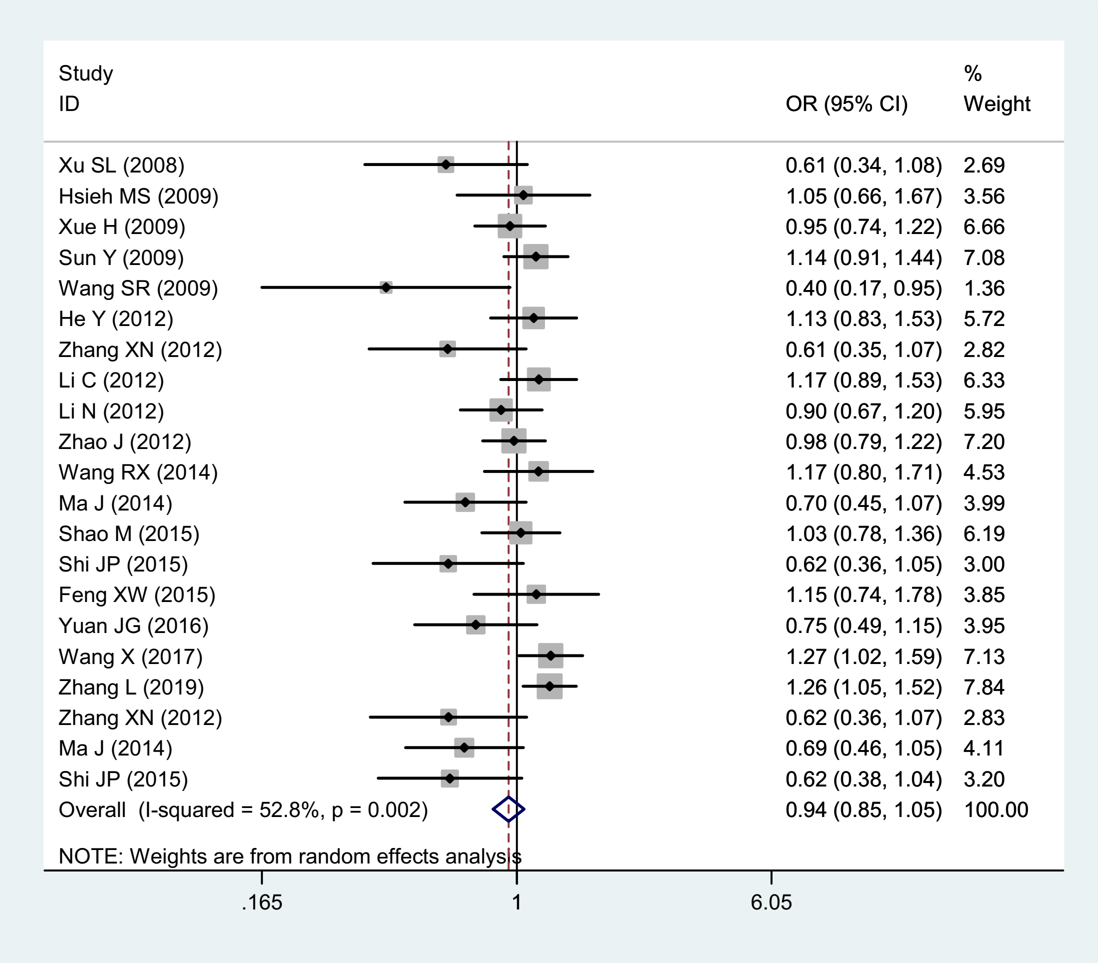

Figure S3: Forest plot for the association between stroke susceptibility and SNP rs2910829 under the dominant model (CT+TT vs CC) (random effects) after excluding those 3 studies on early-onset ischemic stroke (Lin HF(2007), He Y(2013), and Yue X(2019)) and hemorrhagic stroke cases from 2 studies (Xue H(2009) and Wang SR(2009)).

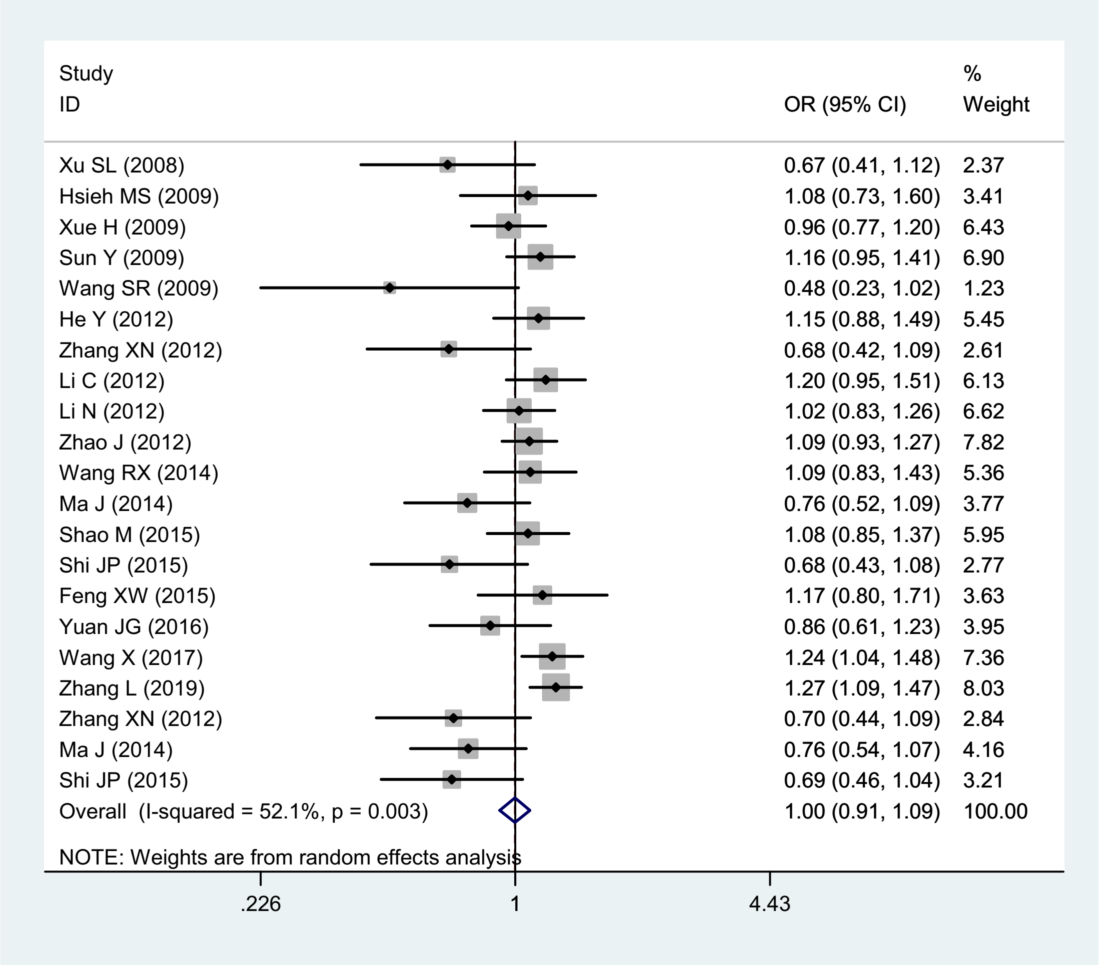

Figure S4: Forest plot for the association between stroke susceptibility and SNP rs2910829 under the additive model (T vs C) (random effects) after excluding those 3 studies on early-onset ischemic stroke (Lin HF(2007), He Y(2013), and Yue X(2019)) and hemorrhagic stroke cases from 2 studies (Xue H(2009) and Wang SR(2009)).

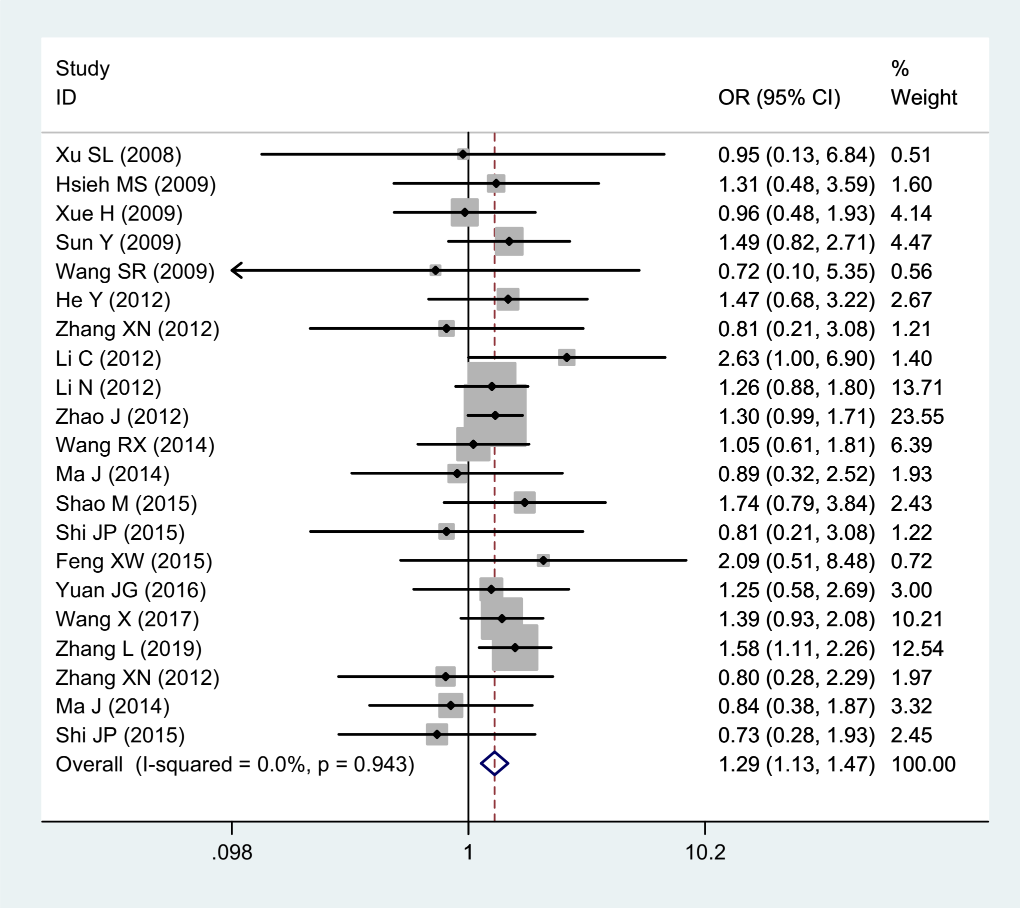

Figure S5: Forest plot for the association between stroke susceptibility and SNP rs2910829 under the recessive model (TT vs CT+CC) (fixed effects) after excluding those 3 studies on early-onset ischemic stroke (Lin HF(2007), He Y(2013), and Yue X(2019)) and hemorrhagic stroke cases from 2 studies (Xue H(2009) and Wang SR(2009)).

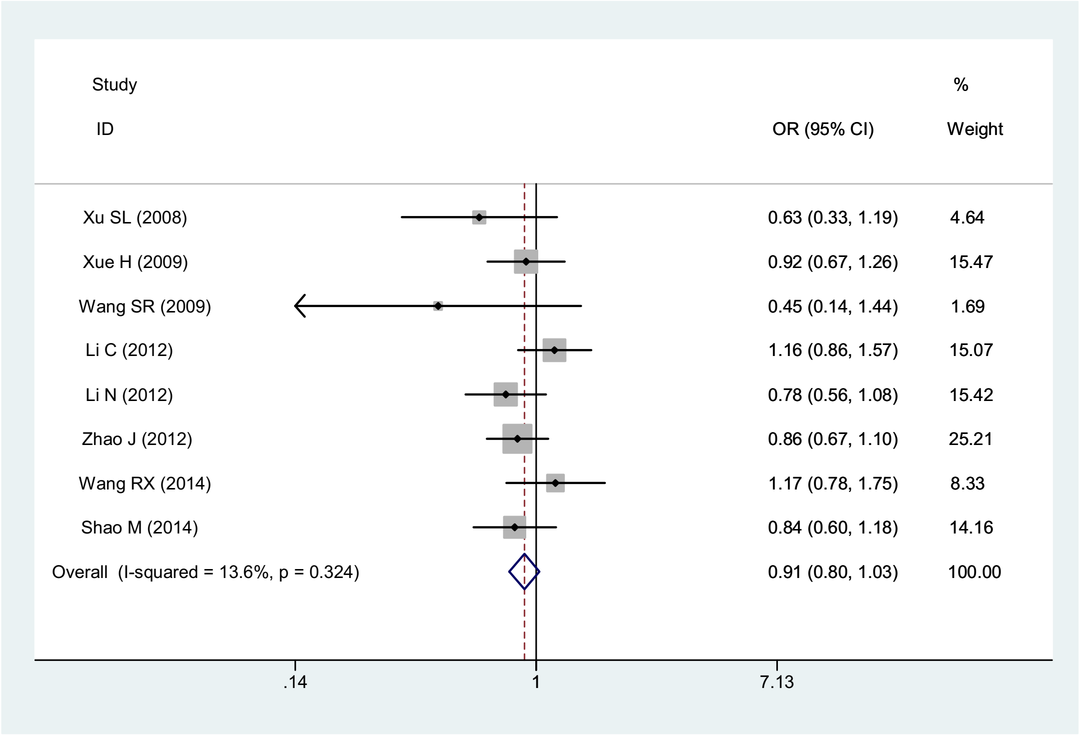

Figure S6: Forest plot for the association between the susceptibility of large artery atherosclerotic stroke and SNP rs2910829 (CT vs CC) (fixed effects).

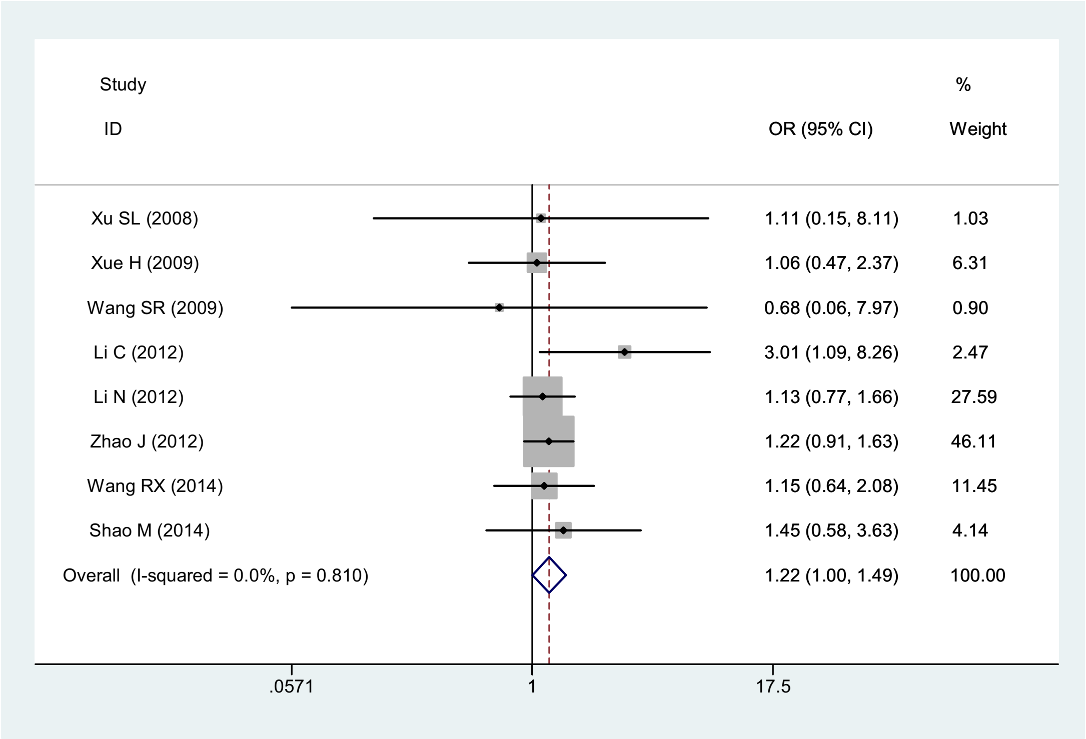

Figure S7: Forest plot for the association between the susceptibility of large artery atherosclerotic stroke and SNP rs2910829 (TT vs CC) (fixed effects).

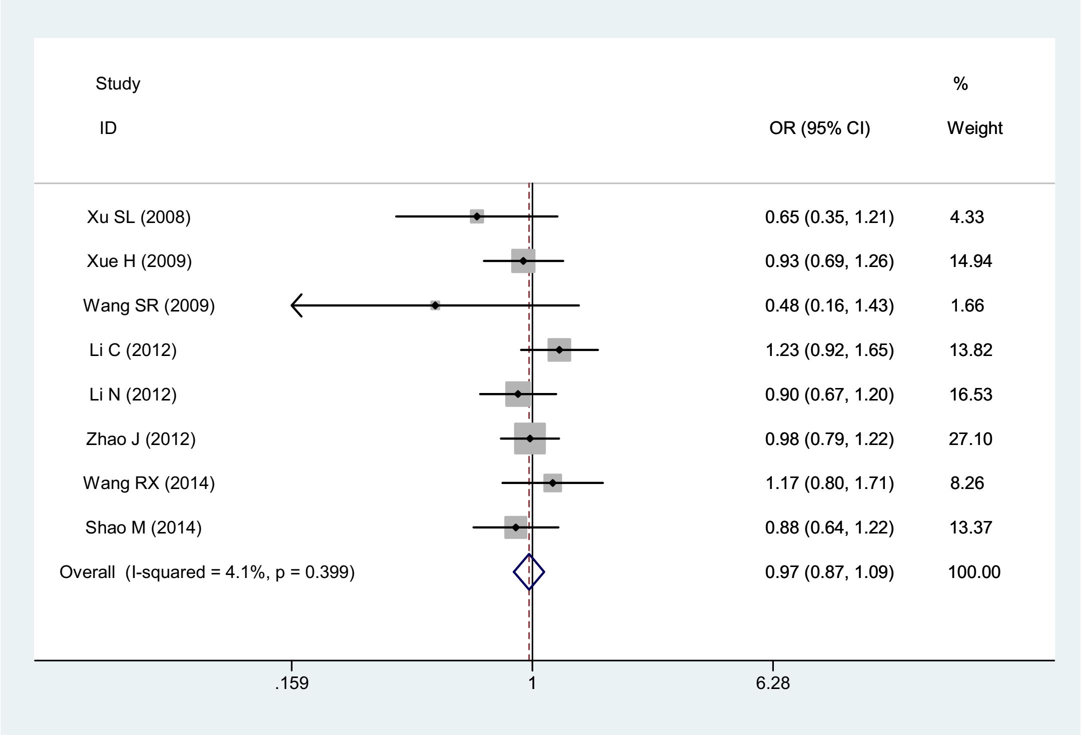

Figure S8: Forest plot for the association between the susceptibility of large artery atherosclerotic stroke and SNP rs2910829 under the dominant model (CT+TT vs CC) (fixed effects).

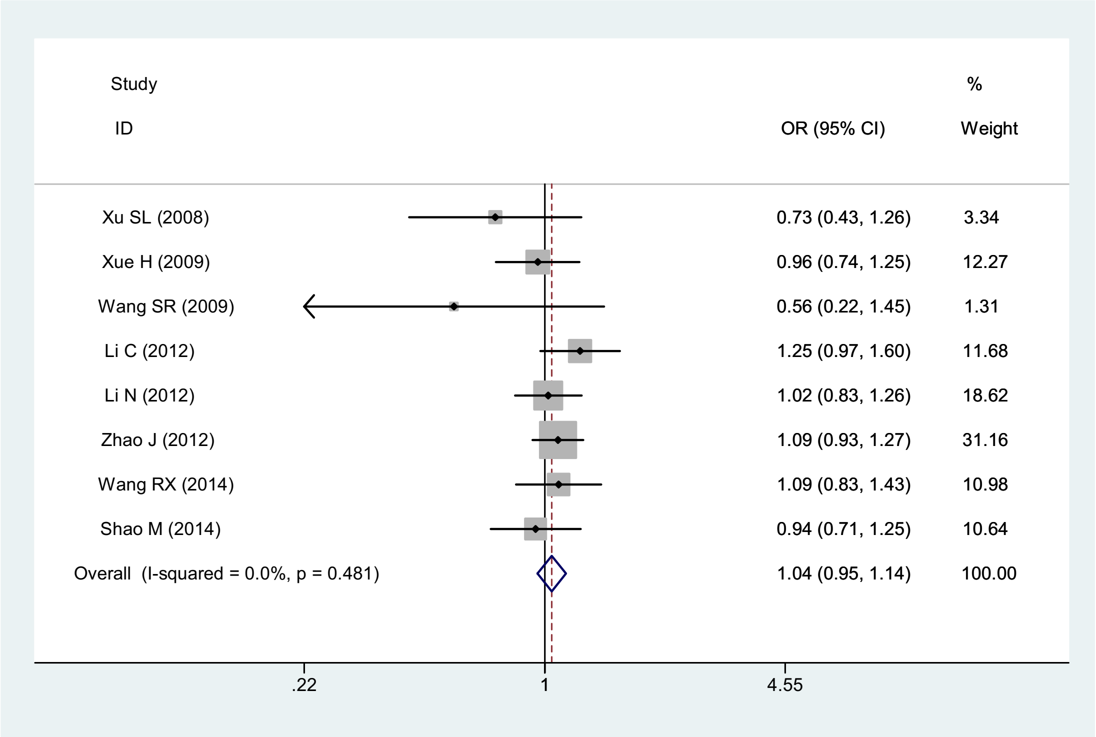

Figure S9: Forest plot for the association between the susceptibility of large artery atherosclerotic stroke and SNP rs2910829 under the additive model (T vs C) (fixed effects).

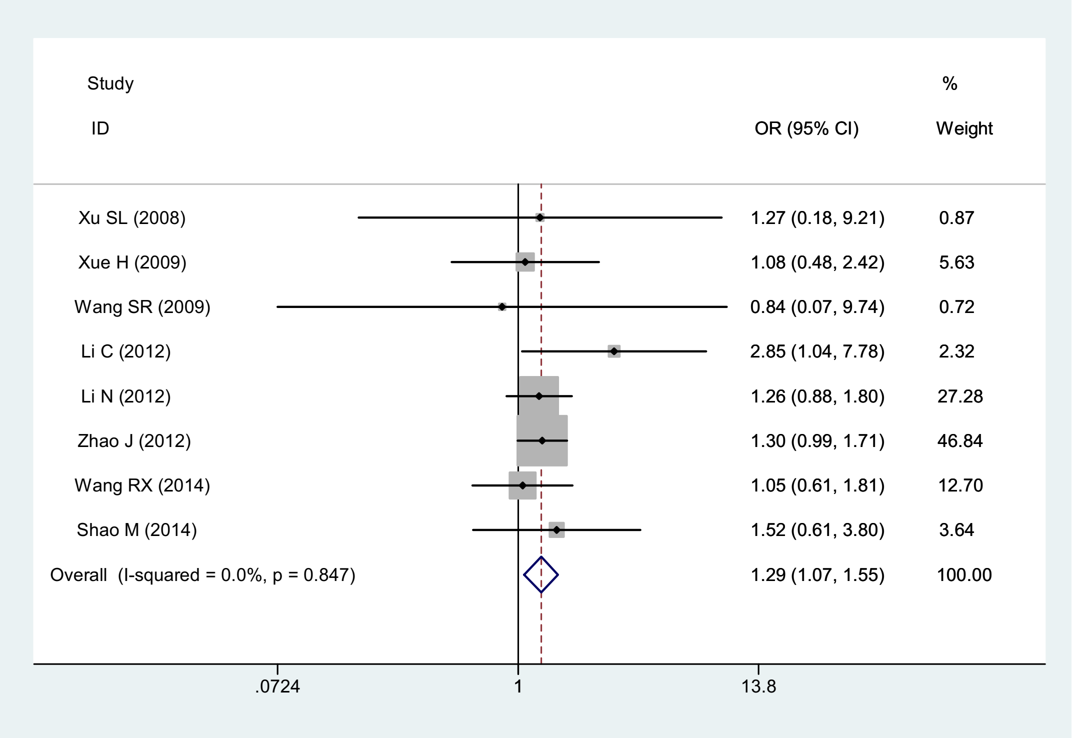

Figure S10: Forest plot for the association between the susceptibility of large artery atherosclerotic stroke and SNP rs2910829 under the recessive model (TT vs CT+CC) (fixed effects).

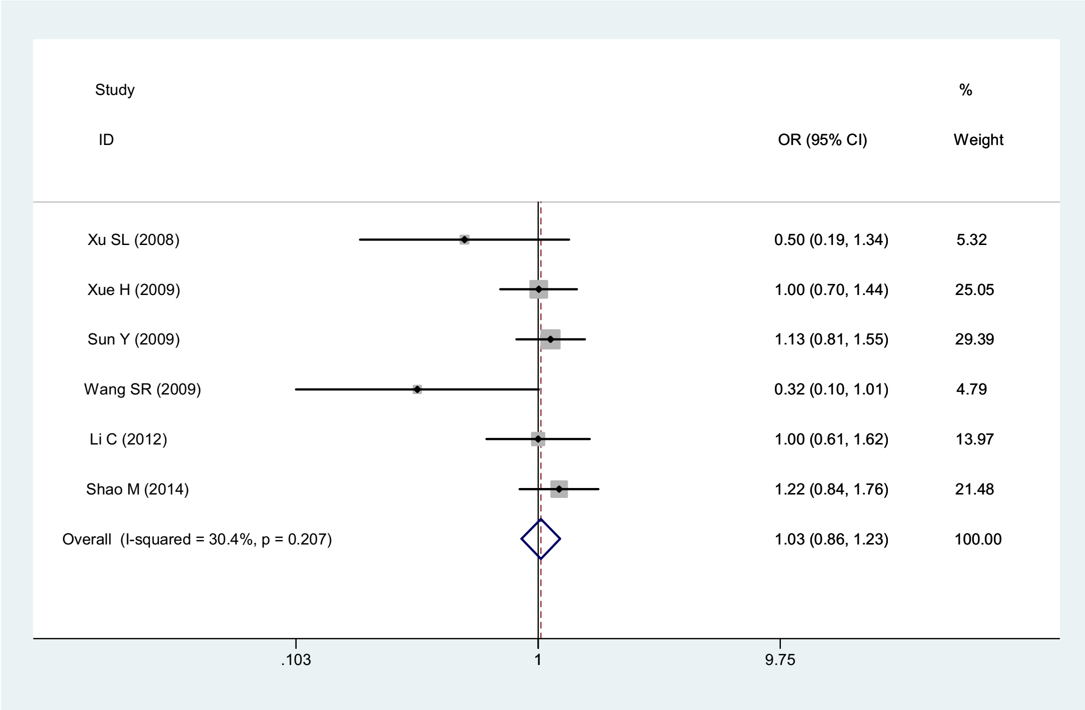

Figure S11: Forest plot for the association between the susceptibility of small vessel stroke and SNP rs2910829 (CT vs CC) (fixed effects).

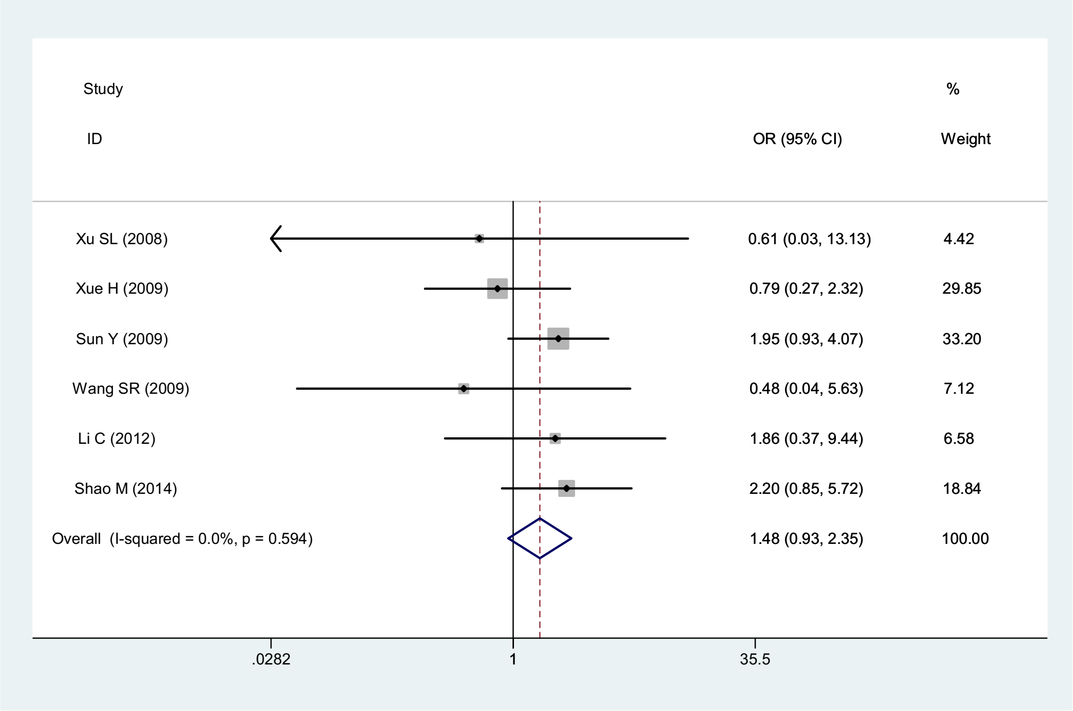

Figure S12: Forest plot for the association between the susceptibility of small vessel stroke and SNP rs2910829 (TT vs CC) (fixed effects).

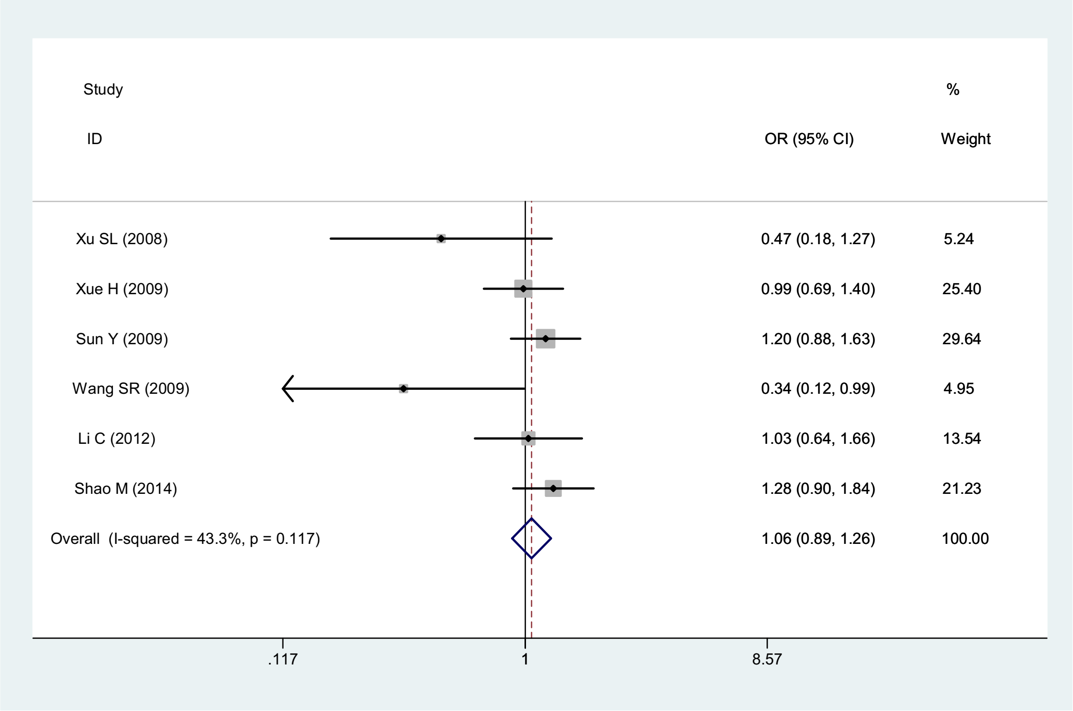

Figure S13: Forest plot for the association between the susceptibility of small vessel stroke and SNP rs2910829 under the dominant model (CT+TT vs CC) (fixed effects).

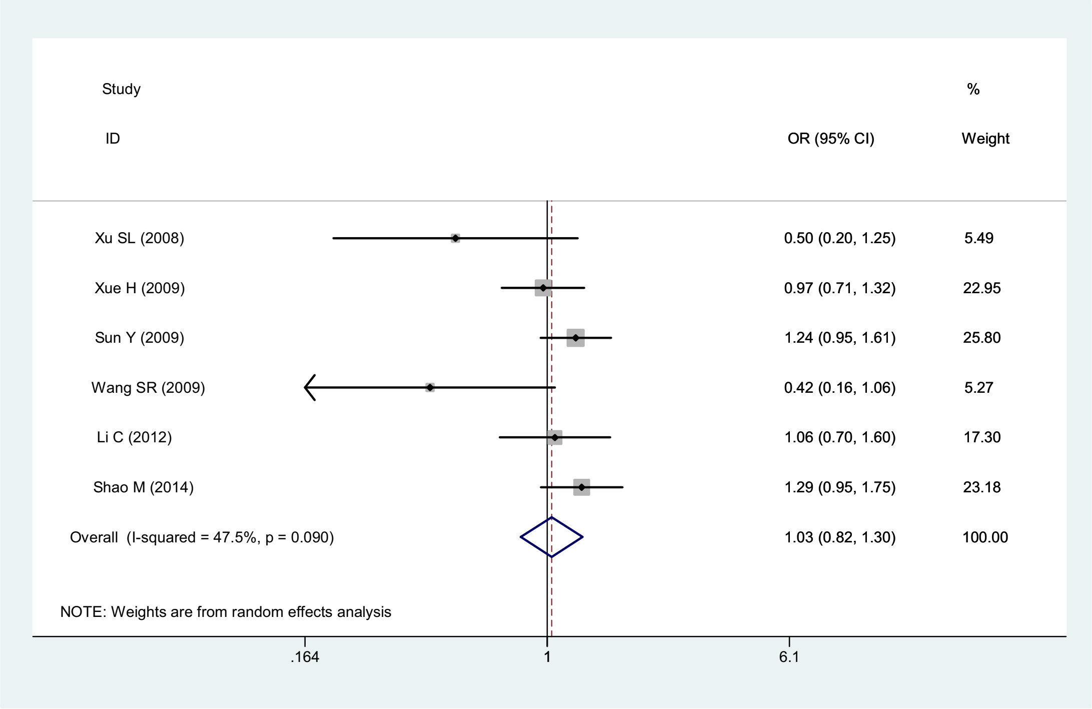

Figure S14: Forest plot for the association between the susceptibility of small vessel stroke and SNP rs2910829 under the additive model (T vs C) (random effects).

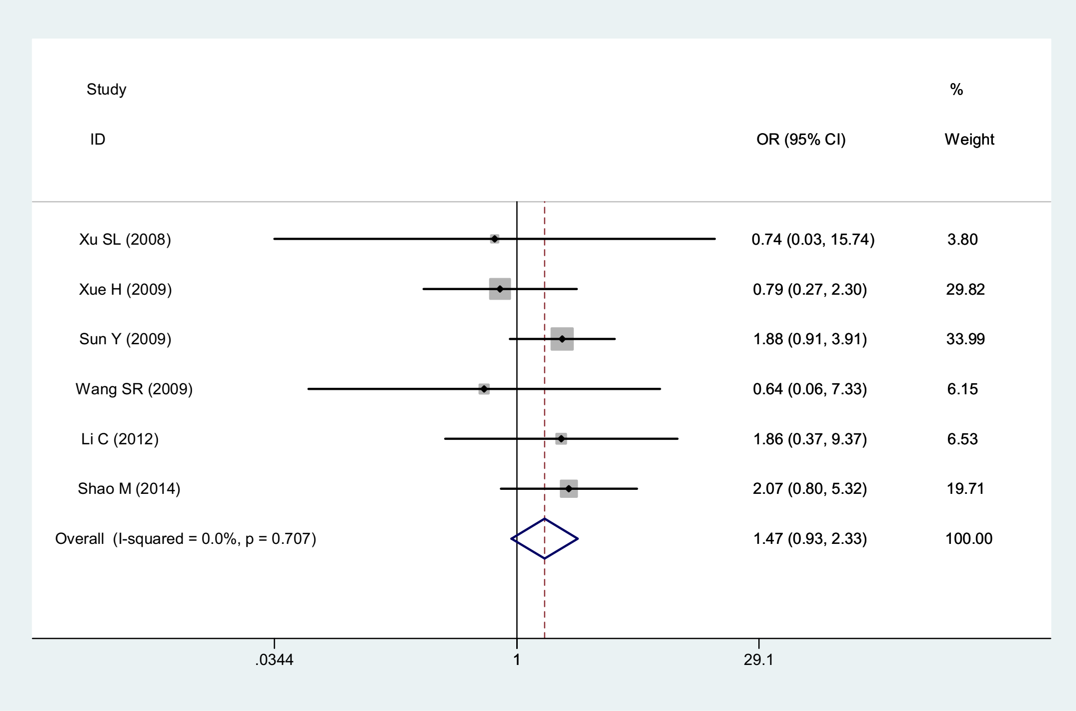

Figure S15: Forest plot for the association between the susceptibility of small vessel stroke and SNP rs2910829 under the recessive model (TT vs CT +CC) (fixed effects).

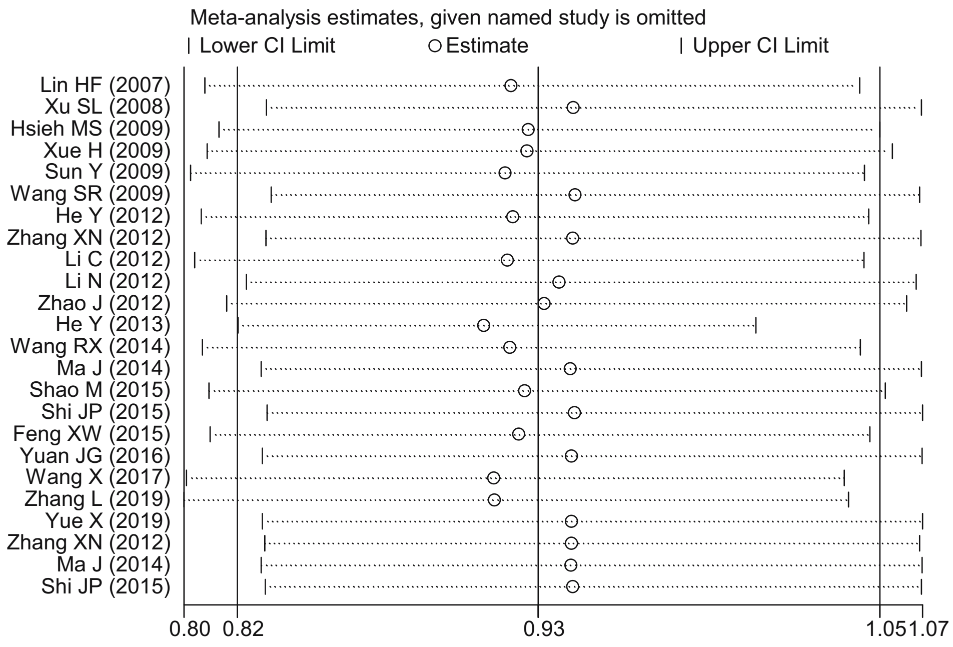

Figure S16: Sensitivity analysis of the summary OR coefficients (CT vs CC). CI, confidence interval; OR, odds ratio.

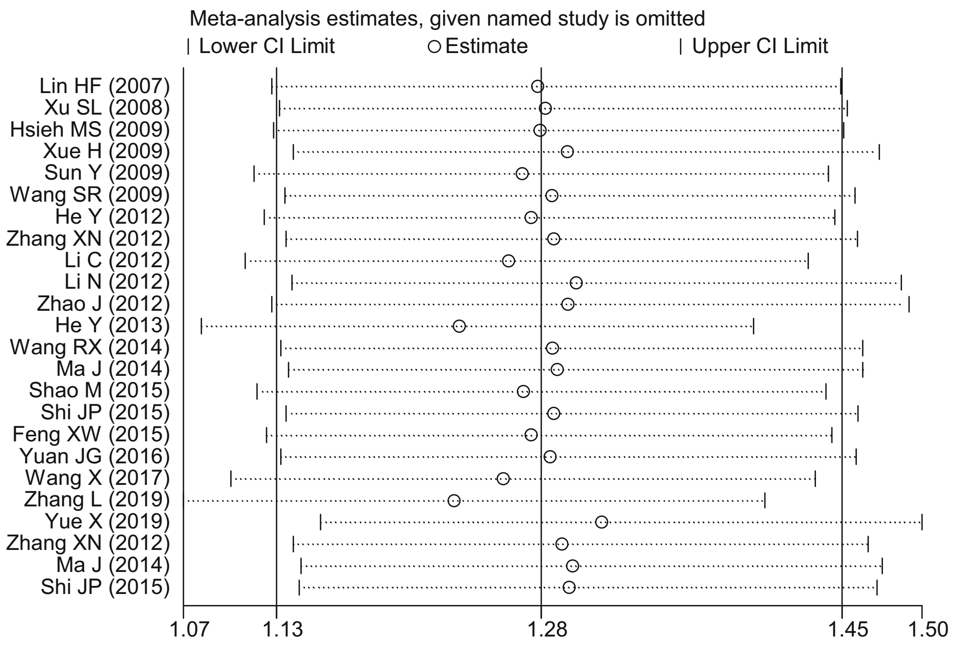

Figure S17: Sensitivity analysis of the summary OR coefficients (TT vs CC). CI, confidence interval; OR, odds ratio.

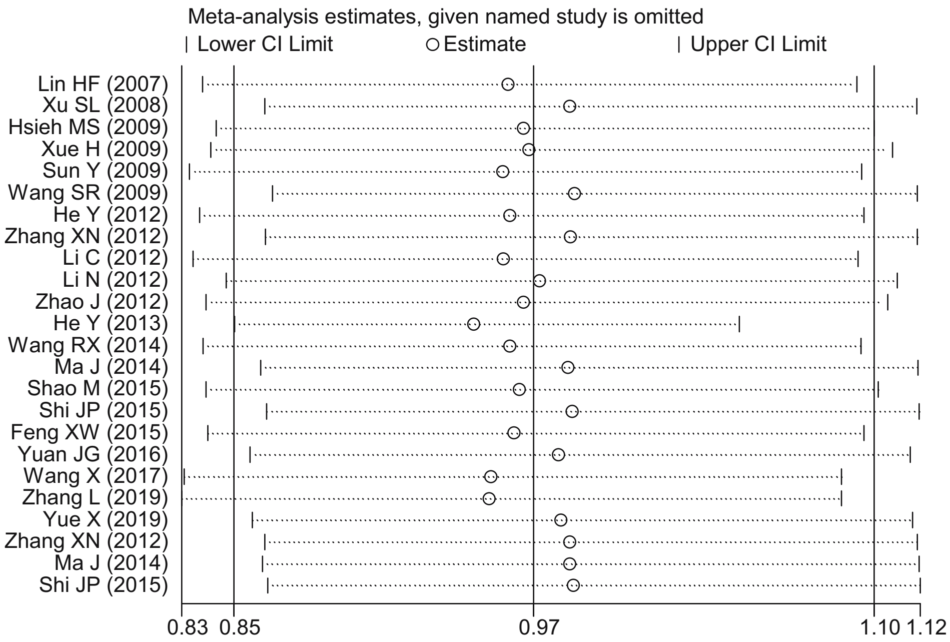

Figure S18: Sensitivity analysis of the summary OR coefficients under the dominant model (CT+TT vs CC). CI, confidence interval; OR, odds ratio.

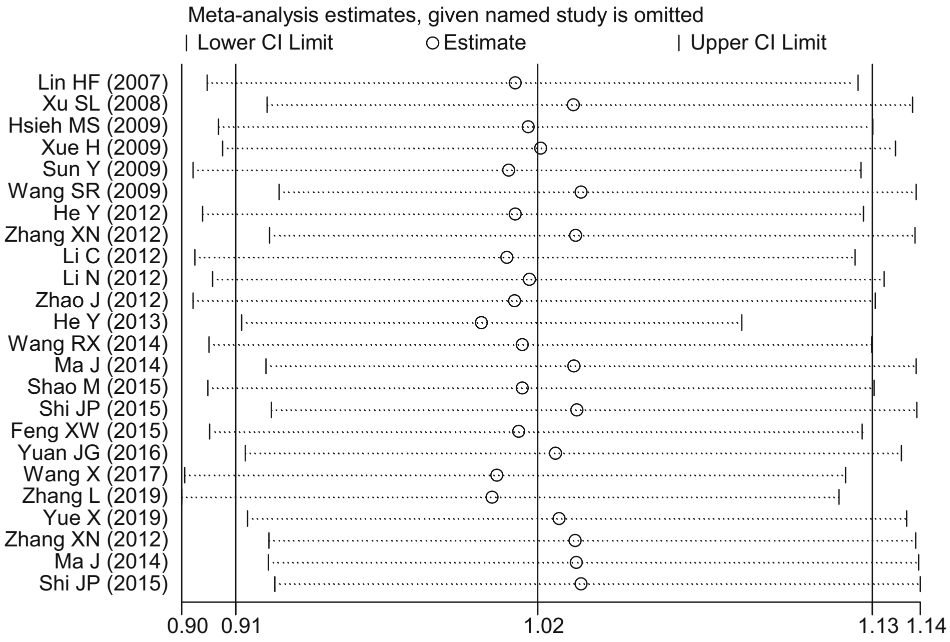

Figure S19: Sensitivity analysis of the summary OR coefficients under the additive model (T vs C). CI, confidence interval; OR, odds ratio.

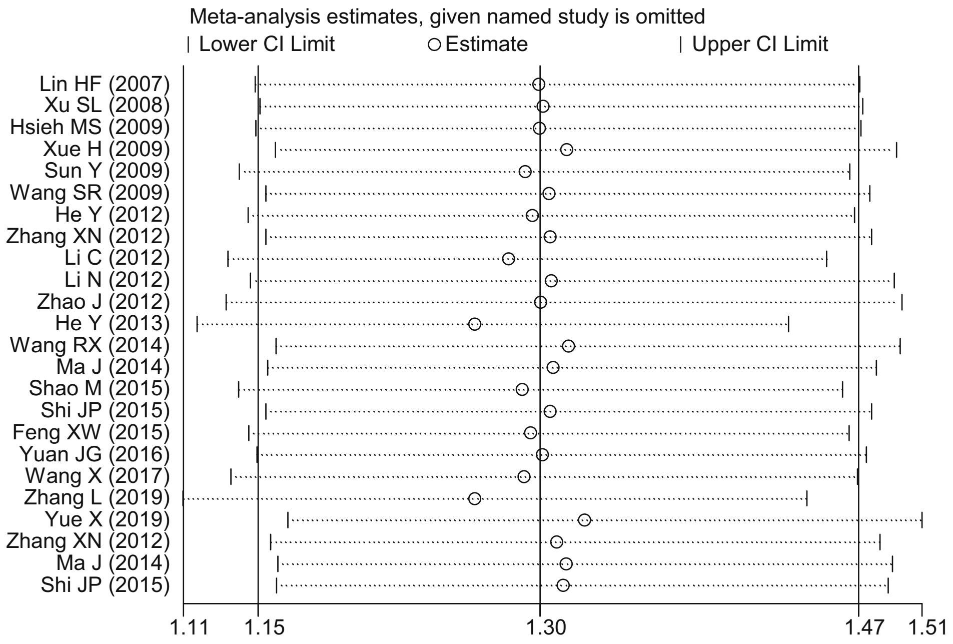

Figure S20: Sensitivity analysis of the summary OR coefficients under the recessive model (TT vs CT+CC). CI, confidence interval; OR, odds ratio.

Table S1: Allele distribution of SNP rs2910829 between stroke cases and controls

First author	Year	Ethnicity	Cases			Controls			
			C	T	MAF	C	T	MAF	HWE
Lin HF	2007	Chinese Han	292	68	0.189	352	68	0.162	0.447
Xu SL	2008	Chinese Han	201	31	0.134	179	41	0.186	0.252
Hsieh MS	2009	Chinese Han	173	43	0.199	455	105	0.188	0.398
Xue H	2009	Chinese Han	1062	216	0.169	1465	309	0.174	0.832
Sun Y	2009	Chinese Han	1060	232	0.180	1280	242	0.159	0.837
Wang SR	2009	Chinese Han	213	31	0.127	69	19	0.216	1.000
He Y	2012	Chinese Han	660	140	0.175	675	125	0.156	0.640
Zhang XN	2012	Chinese Han	197	35	0.151	187	49	0.208	0.961
Li C	2012	Chinese Han	698	182	0.207	798	174	0.179	**0.003**
Li N	2012	Chinese Han	457	285	0.384	461	281	0.379	**2.00×10^{-4}**
Zhao J	2012	Chinese Han	824	540	0.396	746	450	0.376	**3.17×10^{-7}**
He Y	2013	Chinese Han	250	122	0.328	394	70	0.151	0.712
Wang RX	2014	Chinese Han	297	193	0.394	262	156	0.373	0.533
Ma J	2014	Chinese Han	316	62	0.164	308	80	0.206	0.914
Shao M	2015	Chinese Han	751	167	0.182	766	158	0.171	0.250
Shi JP	2015	Chinese Han	214	38	0.151	203	53	0.207	0.793
Feng XW	2015	Chinese Han	265	71	0.211	280	64	0.186	0.137
Yuan JG	2016	Chinese Han	292	74	0.202	283	83	0.227	0.130
Wang X	2017	Chinese Han	863	357	0.293	927	309	0.250	0.171
Zhang L	2019	Chinese Han	1272	490	0.278	1368	416	0.233	0.224
Yue X	2019	Chinese Han	243	143	0.370	238	162	0.405	**1.00×10^{-4}**
Zhang XN	2012	Chinese Uyghur	175	45	0.205	149	55	0.270	0.768
Ma J	2014	Chinese Uyghur	288	80	0.217	268	98	0.268	0.740
Shi JP	2015	Chinese Mongolian	207	53	0.204	178	66	0.270	0.622
Total			7484			7962			

MAF, minor allele frequency; HWE, Hardy-Weinberg equilibrium.

P values significant at $P<0.05$ are shown in bold.

Table S2: Main characteristics of selected studies in the meta-analysis of the association between SNP rs2910829 and ischemic stroke susceptibility

First author	Year	Ethnicity	Sample size		Mean age ± SD(year)		Genotyping method	Matching criteria for controls	Phenotype
			Cases	Controls	Cases	Controls			
Xu SL	2008	Chinese Han	116	110	65.9±12.4	65.1±12.7	PCR-RFLP	Age and gender	LAA and SVD
Hsieh MS	2009	Chinese Han	108	280	70±11	63±23	TaqMan	Age and gender	IS
Xue H	2009	Chinese Han	424	887		60.7±8.2	PCR-RFLP	Age, gender, and geographical region	LAA and SVD
Sun Y	2009	Chinese Han	646	761	73.20±9.41	73.2±7.30	Sanger sequencing	Age, gender, and BMI	IS
Wang SR	2009	Chinese Han	60	44		58.61±17.55	PCR-LDR	Age, gender, and BMI	LAA and SVD
He Y	2012	Chinese Han	400	400	61±10	58±10	PCR-RFLP	Age and gender	IS
Zhang XN	2012	Chinese Han	116	118	61.6±10.6	61.6±10.1	PCR-RFLP	Age and gender	IS
Li C	2012	Chinese Han	440	486	66.58±8.40	66.10±5.18	PCR-RFLP	Age and gender	IS
Li N	2012	Chinese Han	371	371	63.88±7.36	62.87±7.57	PCR-RFLP	Age, gender, and hypercholesterolemia	LAA
Zhao J	2012	Chinese Han	682	598	62.09±9.43	61.84±10.12	PCR-RFLP	Age, gender, and BMI	LAA
Wang RX	2014	Chinese Han	245	209	62.88±8.75	61.21±7.56	PCR-RFLP	Age and gender	LAA
Ma J	2014	Chinese Han	189	194			PCR-RFLP	Age and gender	IS
Shao M	2015	Chinese Han	459	462	68.56±10.97	63.82±9.22	MALDI-TOF	Gender, smoking, and drinking	LAA and SVD
Shi JP	2015	Chinese Han	126	128	60.9±9.7	62.1±9.4	PCR-RFLP	Age and gender	IS
Feng XW	2015	Chinese Han	168	172	65.5±4.7	66.1±5.3	PCR-RFLP	Age, gender, and drinking history	IS
Yuan JG	2016	Chinese Han	183	183	60.2±10.6	60.1±11.3	PCR-RFLP	Age and gender	IS
Wang X	2017	Chinese Han	610	618	65.8±15.2	66.5±16.1	PCR-RFLP	Age and gender	IS
Zhang L	2019	Chinese Han	881	892	64.5±14.7	65.6±15.3	PCR-RFLP	Age, gender, and BMI	IS
Zhang XN	2012	Chinese Uyghur	110	102	61.5±9.9	58.2±9.4	PCR-RFLP	Age and gender	IS
Ma J	2014	Chinese Uyghur	184	183			PCR-RFLP	Age and gender	IS
Shi JP	2015	Chinese Mongolian	130	122	60.87±8.1	59.13±8.9	PCR-RFLP	Age and gender	IS

PCR-RFLP, polymerase chain reaction-restriction fragment length polymorphism; PCR-LDR, polymerase chain reaction ligase detection reaction; MALDI-TOF, matrix-assisted laser desorption/ionization time-of-flight; BMI, body mass index; IS, ischemic stroke; LAA, large artery atherosclerosis; SVD, cerebral small vessel disease.

Table S3: Allele distribution of SNP rs2910829 between ischemic stroke cases and controls

First author	Year	Ethnicity	Cases			Controls			
			C	T	MAF	C	T	MAF	HWE
Xu SL	2008	Chinese Han	201	31	0.134	179	41	0.186	0.252
Hsieh MS	2009	Chinese Han	173	43	0.199	455	105	0.188	0.398
Xue H	2009	Chinese Han	705	143	0.169	1465	309	0.174	0.832
Sun Y	2009	Chinese Han	1060	232	0.180	1280	242	0.159	0.837
Wang SR	2009	Chinese Han	106	14	0.117	69	19	0.216	1.000
He Y	2012	Chinese Han	660	140	0.175	675	125	0.156	0.640
Zhang XN	2012	Chinese Han	197	35	0.151	187	49	0.208	0.961
Li C	2012	Chinese Han	698	182	0.207	798	174	0.179	**0.003**
Li N	2012	Chinese Han	457	285	0.384	461	281	0.379	**2.00×10^{-4}**
Zhao J	2012	Chinese Han	824	540	0.396	746	450	0.376	**3.17×10^{-7}**
Wang RX	2014	Chinese Han	297	193	0.394	262	156	0.373	0.533
Ma J	2014	Chinese Han	316	62	0.164	308	80	0.206	0.914
Shao M	2015	Chinese Han	751	167	0.182	766	158	0.171	0.250
Shi JP	2015	Chinese Han	214	38	0.151	203	53	0.207	0.793
Feng XW	2015	Chinese Han	265	71	0.211	280	64	0.186	0.137
Yuan JG	2016	Chinese Han	292	74	0.202	283	83	0.227	0.130
Wang X	2017	Chinese Han	863	357	0.293	927	309	0.250	0.171
Zhang L	2019	Chinese Han	1272	490	0.278	1368	416	0.233	0.224
Zhang XN	2012	Chinese Uyghur	175	45	0.205	149	55	0.270	0.768
Ma J	2014	Chinese Uyghur	288	80	0.217	268	98	0.268	0.740
Shi JP	2015	Chinese Mongolian	207	53	0.204	178	66	0.270	0.622
Total			6648			7320			

MAF, minor allele frequency; HWE, Hardy-Weinberg equilibrium.

P values significant at $P<0.05$ are shown in bold.

Table S4: Meta-analysis of the association between SNP rs2910829 and ischemic stroke susceptibility

	Pooled OR (95% CI)	P_{OR}	I^2	P_H	Statistical model
CT vs CC	0.91(0.81 – 1.01)	0.084	47.6%	**0.008**	Random
TT vs CC	1.27(1.10 – 1.45)	**0.001**	0.0%	0.673	Fixed
Dominant	0.94(0.85 – 1.05)	0.304	52.8%	**0.002**	Random
Additive	1.00(0.91 – 1.09)	0.949	52.1%	**0.003**	Random
Recessive	1.29(1.13 – 1.47)	**<0.001**	0.0%	0.943	Fixed

P_{OR} and P_H are *P* values for odds ratio and heterogeneity, respectively. P_{OR} values significant at $P < 0.05$ and P_H values significant at $P < 0.10$ are shown in bold.

Table S5: Main characteristics of selected studies in the meta-analysis of the association between SNP rs2910829 and the susceptibility of stroke subtypes

First author	Year	Ethnicity	Sample size		Mean age±SD(year)		Genotyping method	Matching criteria for controls
			Cases	Controls	Cases	Controls		
**Large artery atherosclerotic stroke**								
Xu SL	2008	Chinese Han	87	110	65.3±13.2	65.1±12.7	PCR-RFLP	Age and gender
Xue H	2009	Chinese Han	253	887	62.0±9.1	60.7±8.2	PCR-RFLP	Age, gender, and geographical region
Wang SR	2009	Chinese Han	26	44		58.61±17.55	PCR-LDR	Age, gender, and BMI
Li C	2012	Chinese Han	320	486		66.10±5.18	PCR-RFLP	Age and gender
Li N	2012	Chinese Han	371	371	63.88±7.36	62.87±7.57	PCR-RFLP	Age, gender, and hypercholesterolemia
Zhao J	2012	Chinese Han	682	598	62.09±9.43	61.84±10.12	PCR-RFLP	Age, gender, and BMI
Wang RX	2014	Chinese Han	245	209	62.88±8.75	61.21±7.56	PCR-RFLP	Age and gender
Shao M	2014	Chinese Han	276	462		63.82±9.22	MALDI-TOF	Gender, smoking, and drinking
**Cardioembolic stroke**								
Li C	2012	Chinese Han	32	486		66.10±5.18	PCR-RFLP	Age and gender
**Combined cardioembolic and large artery atherosclerotic stroke**								
Sun Y	2009	Chinese Han	248	761		73.2±7.32	Sanger sequencing	Age, gender, and BMI
Li C	2012	Chinese Han	352	486		66.10±5.18	PCR-RFLP	Age and gender
**Cerebral small vessel disease**								
Xu SL	2008	Chinese Han	29	110	67.9±9.6	65.1±12.7	PCR-RFLP	Age and gender
Xue H	2009	Chinese Han	171	887	60.6±7.2	60.7±8.2	PCR-RFLP	Age, gender, and geographical region
Sun Y	2009	Chinese Han	248	761		73.2±7.32	Sanger sequencing	Age, gender, and BMI
Wang SR	2009	Chinese Han	34	44		58.61±17.55	PCR-LDR	Age, gender, and BMI
Li C	2012	Chinese Han	88	486		66.10±5.18	PCR-RFLP	Age and gender
Shao M	2014	Chinese Han	183	462		63.82±9.22	MALDI-TOF	Gender, smoking, and drinking
**Hemorrhagic stroke**								
Xue H	2009	Chinese Han	215	887	59.9±9.9	60.7±8.2	PCR-RFLP	Age, gender, and geographical region
Wang SR	2009	Chinese Han	62	44		58.61±17.55	PCR-LDR	Age, gender, and BMI

PCR-RFLP, polymerase chain reaction-restriction fragment length polymorphism; PCR-LDR, polymerase chain reaction ligase detection reaction; MALDI-TOF, matrix-assisted laser desorption/ionization time-of-flight; BMI, body mass index.

Table S6: Allele distribution of SNP rs2910829 between cases and controls of the stroke subtypes

First author	Year	Ethnicity	Cases			Controls			
			C	T	MAF	C	T	MAF	HWE
**Large artery atherosclerotic stroke**									
Xu SL	2008	Chinese Han	149	25	0.144	179	41	0.186	0.252
Xue H	2009	Chinese Han	421	85	0.168	1465	309	0.174	0.832
Wang SR	2009	Chinese Han	45	7	0.135	69	19	0.216	1.000
Li C	2012	Chinese Han	503	137	0.214	798	174	0.179	**0.003**
Li N	2012	Chinese Han	457	285	0.384	461	281	0.379	$\mathbf{2.00 \times 10^{-4}}$
Zhao J	2012	Chinese Han	824	540	0.396	746	450	0.376	$\mathbf{3.17 \times 10^{-7}}$
Wang RX	2014	Chinese Han	297	193	0.394	262	156	0.373	0.533
Shao M	2014	Chinese Han	462	90	0.163	766	158	0.171	0.250
Total			2260			3167			
**Cardioembolic stroke**									
Li C	2012		52	12	0.188	798	174	0.179	**0.003**
**Combined cardioembolic and large artery atherosclerotic stroke**									
Sun Y	2009	Chinese Han	401	95	0.192	1280	242	0.159	0.837
Li C	2012	Chinese Han	555	149	0.212	798	174	0.179	**0.003**
Total			600			1247			
**Cerebral small vessel disease**									
Xu SL	2008	Chinese Han	52	6	0.103	179	41	0.186	0.252
Xue H	2009	Chinese Han	284	58	0.170	1465	309	0.174	0.832
Sun Y	2009	Chinese Han	402	94	0.190	1280	242	0.159	0.837
Wang SR	2009	Chinese Han	61	7	0.103	69	19	0.216	1.000
Li C	2012	Chinese Han	143	33	0.188	798	174	0.179	**0.003**
Shao M	2014	Chinese Han	289	77	0.210	766	158	0.171	0.250
Total			753			2750			
**Hemorrhagic stroke**									
Xue H	2009	Chinese Han	357	73	0.170	1465	309	0.174	0.832
Wang SR	2009	Chinese Han	107	17	0.137	69	19	0.216	1.000
Total			277			931			

MAF, minor allele frequency; HWE, Hardy-Weinberg equilibrium.

P values significant at $P<0.05$ are shown in bold.

Table S7: Meta-analysis of the association between SNP rs2910829 and the susceptibility of large artery atherosclerotic stroke

	Pooled OR (95% CI)	P_{OR}	I^2	P_H	Statistical model
CT vs CC	0.91(0.80 – 1.03)	0.128	13.6%	0.324	Fixed
TT vs CC	1.22(1.00 – 1.49)	**0.045**	0.0%	0.810	Fixed
Dominant	0.97(0.87 – 1.09)	0.653	4.1%	0.399	Fixed
Additive	1.05(0.96 – 1.14)	0.342	0.0%	0.481	Fixed
Recessive	1.29(1.07 – 1.55)	**0.008**	0.0%	0.847	Fixed

P_{OR} and P_H are P values for odds ratio and heterogeneity, respectively. P_{OR} values significant at $P < 0.05$ and P_H values significant at $P < 0.10$ are shown in bold.

Table S8: Meta-analysis of the association between SNP rs2910829 and the susceptibility of small vessel stroke

	Pooled OR (95% CI)	P_{OR}	I^2	P_H	Statistical model
CT vs CC	1.03(0.86 – 1.23)	0.785	30.4%	0.207	Fixed
TT vs CC	1.48(0.93 – 2.36)	0.095	0.0%	0.594	Fixed
Dominant	1.06(0.89 – 1.26)	0.514	43.3%	0.117	Fixed
Additive	1.03(0.82 – 1.30)	0.780	47.5%	**0.090**	Random
Recessive	1.47(0.93 – 2.33)	0.099	0.0%	0.707	Fixed

P_{OR} and P_H are P values for odds ratio and heterogeneity, respectively. P_{OR} values significant at $P < 0.05$ and P_H values significant at $P < 0.10$ are shown in bold.

Table S9: Meta-analysis of the association between SNP rs2910829 and stroke susceptibility after excluding the HWE-violating studies

	Pooled OR (95% CI)	P_{OR}	I^2	P_H	Statistical model
CT vs CC	0.94(0.81 – 1.09)	0.418	67.0%	<**0.001**	Random
TT vs CC	1.35(1.15 – 1.60)	<**0.001**	26.7%	0.132	Fixed
Dominant	0.97(0.83 – 1.13)	0.652	73.0%	<**0.001**	Random
Additive	1.00(0.88 – 1.14)	0.995	69.6%	<**0.001**	Random
Recessive	1.32(1.13 – 1.56)	**0.001**	0.0%	0.624	Fixed

P_{OR} and P_H are P values for odds ratio and heterogeneity, respectively.
P_{OR} values significant at $P<0.05$ and P_H values significant at $P<0.10$ are shown in bold.

Table S10: Functional annotations for SNP rs2910829 (SNP87) and variants in strong LD with rs2910829 (defined as $r^2 \geq 0.8$ with rs2910829 in the East Asian population) using HaploReg v4.1

	Position (hg38)	LD		Variant	Ref	Alt	Frequency				GERP cons	SiPhy cons	Promoter histone marks	Enhancer histone marks	DNase	Proteins bound	Motifs changed	NHGRI/EBI GWAS hits	GRASP QTL hits	Selected eQTLhits	GENCODE genes	dbSNP func annot
		r^2	D'				AFR	AMR	ASN	EUR												
1	60155192	0.8	0.97	rs10939837	C	T	0.48	0.54	0.16	0.56							10 altered motifs				PDE4D	intronic
2	60157997	0.8	0.97	rs6449458	C	G	0.49	0.55	0.16	0.56							Pou2f2				PDE4D	intronic
3	60174072	1	1	**rs2910829**	G	A	0.48	0.55	0.19	0.57							Ik-2,SIX5,STAT				PDE4D	intronic

SNP rs2910829 is shown in bold.

LD, linkage disequilibrium; Ref, reference; Alt, alternate; AFR, African (YRI, LWK, ASW); AMR, American (MXL, CLM, PUR); ASN, East Asian (CHB, JPT, CHS); EUR, European (CEU, TSI, GBR, FIN, IBS); GERP cons, sequence constraint by GERP; SiPhy cons, sequence constraint by SiPhy; DNase, deoxyribonuclease; GWAS, genome-wide association study; QTL, quantitative trait locus; eQTL, expression quantitative trait locus; func annot, functional annotation.

Table S11: Functional annotations for SNP rs2910829 (SNP87) and variants in strong LD with rs2910829 (defined as $r^2 \geq 0.8$ with rs2910829 in the East Asian population) using RegulomeDB v2.1 in GRCh38 assembly

	dbSNP IDs	Rank	Score	DNase	Protein binding	eQTL		Motifs
						Biosample	Target genes	
1	rs10939837	1f	0.24002	brain		esophagus muscularis mucosa	PART1 (ENSG00000152931.7)	BSX,DLX3,DLX4,DLX6,EN2,LHX1,MSX1,MSX2
						frontal cortex	CTC-436P18.5 (ENSG00000276945.1)	
2	rs6449458	7	0.51392			frontal cortex	CTC-436P18.5 (ENSG00000276945.1)	
3	**rs2910829**	6	0.78165			esophagus muscularis mucosa	PART1 (ENSG00000152931.7)	THAP11
						frontal cortex	CTC-436P18.5 (ENSG00000276945.1)	

SNP rs2910829 is shown in bold.

LD, linkage disequilibrium; DNase, deoxyribonuclease; eQTL, expression quantitative trait locus.

Table S12: Meta-analysis reports involving the role of SNP rs2910829 in stroke by 2022

No.	Reference	Population	Phenotype	No. of studies included	Sample size		Ethnicity subgroup analysis	Stroke subtype analysis	Nature of risk association
					Cases	Controls			
1	Staton et al. (2006)	NA	Stroke	9	3,808	4,377	No	No	Significant association found
2	Bevan et al. (2008)	Caucasians (≈93%) and non-Caucasians	IS	7 (6 in Caucasians)	3,653 (3,381 in Caucasians)	4,442 (4,168 in Caucasians)	Yes	Yes	No association found in IS or its subtypes; and no association found in Caucasians
3	Xu et al. (2010)	Asians	IS	4	2,308	4,133	No	No	No association found
4	Liu et al. (2011)	Asians, Caucasians, and other ethnicities	IS	14	NA	NA	Yes	No	No association found
5	Yoon et al. (2011)	Asians, Caucasians, and other ethnicities	IS	11 (5 in Asians)	6,282 (2,512 in Asians)	9,949 (5,908 in Asians)	Yes	No	No association found
6	Yadav et al. (2013)	South Asians	IS	2	450	500	No	No	No association found
7	Liang et al. (2015)	Asians and Caucasians	IS	18 (11 in Asians)	8,363 (3,951 in Asians)	12,223 (7,599 in Asians)	Yes	No	No association found
8	Wu et al. (2017)	Asians, Caucasians, and other ethnicities	IS	18 (7 in Asians)	NA	NA	Yes	No	No association found
9	Wei et al. (2017)	Asians and Caucasians	IS	26 (16 in Asians)	10,529 (5,724 in Asians)	12,667 (7,731 in Asians)	Yes	No	Significant associations found in Asians but not in Caucasians and the overall population
10	Current meta-analysis	Chinese	Stroke	24	7,484	7,962	No	Yes	Significant association found in stroke and its subtypes

No., number; NA, not available; IS, ischemic stroke.
